# China’s carbon-neutral policies will reduce short-term PM_2.5_-associated excess incidence of cardiovascular diseases

**DOI:** 10.1016/j.oneear.2024.01.006

**Published:** 2024-03-15

**Authors:** Jie Ban, Jing Cheng, Can Zhang, Kailai Lu, Zhen Zhou, Zhao Liu, Yidan Chen, Can Wang, Wenjia Cai, Peng Gong, Yong Luo, Dan Tong, Jianlin Hu, Xinbiao Guo, Junwei Hao, Tiantian Li

**Affiliations:** 1 National Key Laboratory of Intelligent Tracking and Forecasting for Infectious Diseases, National Institute of Environmental Health, Chinese Center for Disease Control and Prevention, Beijing 100021, China; 2 China CDC Key Laboratory of Environment and Population Health, National Institute of Environmental Health, Chinese Center for Disease Control and Prevention, Beijing 100021, China; 3 Collaborative Innovation Center of Atmospheric Environment and Equipment Technology, Jiangsu Key Laboratory of Atmospheric Environment Monitoring and Pollution Control (AEMPC), Nanjing University of Information Science & Technology, Nanjing 210044, China; 4 Department of Earth System Science, Ministry of Education Key Laboratory for Earth System Modeling, Institute for Global Change Studies, Tsinghua University, Beijing 100084, China; 5 School of Linkong Economics and Management, Beijing Institute of Economics and Management, Beijing 100024, China; 6 State Key Joint Laboratory of Environment Simulation and Pollution Control (SKLESPC), School of Environment, Tsinghua University, Beijing 100084, China; 7 Department of Earth Sciences and Geography, University of Hong Kong, Hong Kong Special Administrative Region 999077, China; 8 Department of Occupational and Environmental Health Sciences, School of Public Health, Peking University, No. 38 Xueyuan Road, Beijing 100191, China; 9 Department of Neurology, Xuanwu Hospital, Capital Medical University, National Center for Neurological Disorders, No. 45 Changchun Street, Xicheng District, Beijing 100053, China; 10 Key Laboratory for Neurodegenerative Diseases of Ministry of Education, No. 45 Changchun Street, Xicheng District, Beijing 100053, China; 11 Beijing Municipal Geriatric Medical Research Center, No. 45 Changchun Street, Xicheng District, Beijing 100053, China

**Keywords:** ambient PM_2.5_, short-term exposure, incidence, cardiovascular disease, carbon neutral, projection, health benefit

## Abstract

China’s carbon-neutral target could have benefits for ambient fine particulate matter (PM_2.5_)-associated mortality. Although previous studies have researched such benefits, the potential impact on cardiovascular disease incidence burden is yet to be investigated thoroughly. Here, we first estimate the association between short-term PM_2.5_ exposure and the incidence of stroke and coronary heart disease (CHD) via a case-crossover study before projecting future changes in short-term PM_2.5_-associated excess incidence across China from 2025 to 2060 under three different emission scenarios. We find that, compared to the 2015–2020 baseline, average PM_2.5_ concentrations nationwide in 2060 under SSP119 (an approximation of a carbon-neutral scenario) are projected to decrease by 81.07%. The short-term PM_2.5_-related excess incidence of stroke and CHD is projected to be reduced to 3,352 cases (95% confidence interval: 939, 5,738)—compared with 34,485 cases under a medium-emissions scenario (SSP245)—and is expected to be accompanied by a 95% reduction in the related economic burden. China’s carbon-neutral policies are likely to bring health benefits for cardiovascular disease by reducing short-term PM_2.5_-related incidence burden.

## Highlights

• PM_2.5_ of SSP119 in 2060 will drop by 81.07% compared to the average level in 2015–2020
• Short-term PM_2.5_-related excess incidence of cardiovascular diseases will be reduced to 3,352
• Emission control could be the key driving factor for the related health benefits

## Science for society

Ambient fine particulate matter (PM_2.5_) can penetrate deep into the cardiorespiratory system and trigger cardiovascular diseases. In 2019, ambient PM_2.5_ caused almost 1 million premature cardiovascular deaths in China. China’s commitment to attaining carbon neutrality by 2060 may help reduce this burden given the potential decrease in PM_2.5_ as a consequence of carbon dioxide emissions control. However, the impact of China’s carbon neutrality target on PM_2.5_-associated incidence of cardiovascular diseases remains unclear due to a lack of sophisticated analysis. Projections generated in this study indicate that China’s carbon neutrality policies could alleviate the short-term PM_2.5_-associated excess incidence of cardiovascular disease by 90.3% in 2060 relative to policies in place between 2015 and 2020. The implementation of continuous, region-specific emission reduction strategies by policymakers is crucial to achieve effective co-control of local PM_2.5_ pollution and associated public health benefits.

## Graphical abstract

**Figure:**
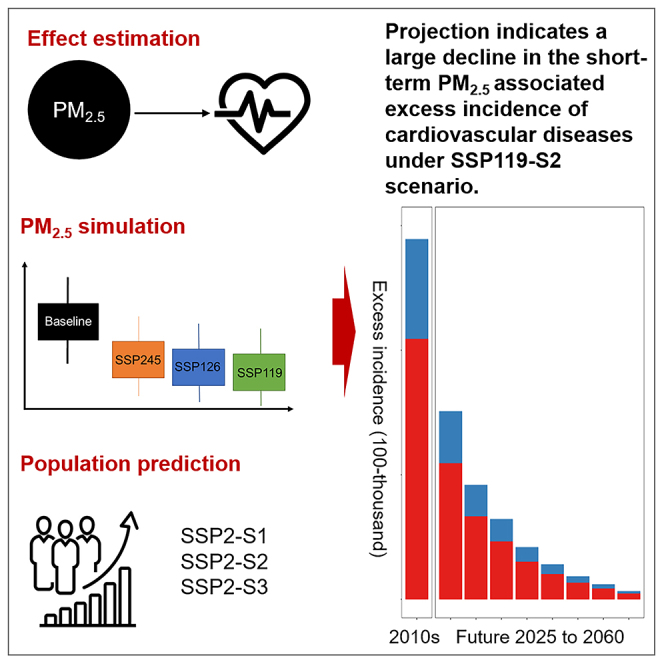

## Teaser

China’s commitment to carbon neutrality may alter PM_2.5_ pollution and its associated disease burdens. Projecting forward to 2060 under different carbon mitigation targets, this comprehensive projection study finds substantial reduction in annual average PM_2.5_ levels and the short-term PM_2.5_-associated excess incidence of cardiovascular diseases under SSP119 (approximately carbon-neutral scenario) in 2060 compared to the 2015–2020 baseline. Emission control is the dominant contributor driving these declines in different regions. China’s carbon-neutral policies will bring health benefits and potential savings in healthcare expenditures.

## Introduction

Ambient fine particulate matter (PM_2.5_) has caused 1,423 thousand deaths in China, with over 60% attributable to cardiovascular deaths.^1^^,^^2^^,^^3^ Although a series of clean air policies are reported to have resulted in considerable improvement in China’s air quality,^4^^,^^5^^,^^6^^,^^7^^,^^8^^,^^9^ stronger policies are still required to substantially reduce the concentration of PM_2.5_ to protect public health in the country^10^ and to align with the World Health Organization’s (WHO) newly announced global air quality guidelines (AQG). In September 2020, a carbon-neutral target was launched by the Chinese government to mitigate the effects of climate change. This target includes the objective of peaking CO_2_ emissions before 2030 and achieving carbon neutrality before 2060. Considering that the causes and solutions of air pollution and climate change are closely linked,^11^ mitigating climate change may also bring air quality improvement and have potential benefits for the health risks associated with ambient PM_2.5_.^7^^,^^12^ One of the associated health risks, cardiovascular diseases, is rising in incidence in China^13^^,^^14^^,^^15^ and thus requires particular attention. However, to assess the potential effectiveness of China’s coordinated governance of climate change and air pollution, we must first determine the incidence of cardiovascular diseases associated with ambient PM_2.5_ and project how these might change in the future.

Previous studies have projected the future PM_2.5_-associated mortality under different scenarios of climate control and air pollution policies in China^7^^,^^16^^,^^17^^,^^18^^,^^19^^,^^20^^,^^21^ and worldwide^22^; however, few of them have focused on the health burden related to short-term PM_2.5_ under carbon-neutral-target scenarios. It thus remains unclear how carbon-neutral-target-related policies may impact the PM_2.5_-related disease burden in China. Furthermore, due to the lack of robust exposure-response relationships,^23^^,^^24^^,^^25^^,^^26^^,^^27^^,^^28^^,^^29^^,^^30^^,^^31^ especially regarding the association between the incidence of cardiovascular diseases and short-term PM_2.5_ exposure, previous studies have mostly focused on projecting the mortality burden related to ambient PM_2.5_ rather than the incidence burden. Therefore, the future trend of the incidence risk of cardiovascular diseases related to PM_2.5_ is still not well understood.

Here, we take a three-step approach to project future short-term PM_2.5_-associated excess incidence of cardiovascular diseases (Figure S1): first we apply a case-crossover study to estimate the exposure-response relationship between daily PM_2.5_ exposure and incidence of stroke and coronary heart disease (CHD); second, we use an air quality modeling system to simulate the future daily PM_2.5_ concentration from 2025 to 2060 under three emission scenarios (Table S1)—SSP245 (current-situation scenario), SSP126 (carbon-peak scenario), and SSP119 (approximate carbon-neutral scenario); and finally, we assess the short-term PM_2.5_-associated excess incidence of CHD and stroke for the baseline period of 2015–2020 and future periods and explore the contribution of driving factors. We find that, compared to the 2015–2020 baseline, annual average PM_2.5_ concentrations nationwide in 2060 under SSP119 (approximately carbon-neutral scenario) are projected to decrease by 81.07%. The short-term PM_2.5_-related excess incidence of the two diseases will be reduced to 3,352 (95% confidence interval [CI]: 939, 5,738), far less than 34,485 (95% CI: 9,662, 59,022) under SSP245. Related healthcare expenditure is projected to reduce by more than 95% under SSP119. The continuous emission control under China’s carbon-neutral policies could play a critical role in bringing large health benefits and saving healthcare expenditure.

## Results

### Association between PM_2.5_ and disease incidence

A total of 129,568 CHD and 143,552 stroke cases were included in this study (Figure S2). Overall, the average age of cases was around 70 years old, and the incidence was higher in males than in females (see Table 1). Our exposure data distribution presented heterogeneity in both air pollution and meteorological conditions (see Table S2; Figure S2).

**Table 1:** Summary description on incidence data between 2013 and 2018

	CHD	Stroke
No. of cases	129,568	143,552
Age (mean, years old)	70.9	71.9
Sex (no. [%])		
Female	57,393 (44.3)	65,704 (45.8)
Male	72,175 (55.7)	77,846 (54.2)
Incidence rate (per 100,000 people)	126.82	375.83

**Figure 1:**
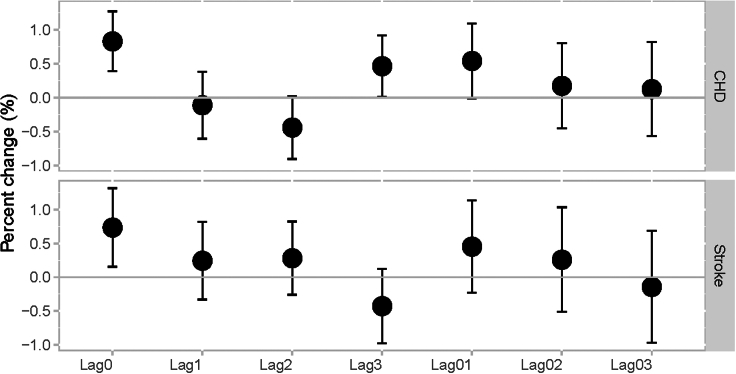
Percent change in incidence risk associated with each 10 μg/m^3^ increase of PM_2.5_ on lag0 Error bars indicate the 95% confidence intervals for the estimated percent change.

Figure 1 indicated that the current day exposure of PM_2.5_ (lag0) was associated with the highest increase in CHD and stroke incidence. With every 10 μg/m^3^ rise in the lag0 day’s PM_2.5_, there was an associated increase of 0.83% (95% CI: 0.39%, 1.27%) in the acute incidence of CHD and a 0.73% (95% CI: 0.15%, 1.31%) increase in stroke incidence. Figure 1 presents a gradual decline in the associations from lag1 to lag 3, while the association between CHD incidence and exposure on lag3 day reached statistical significance, which suggested that the triggering effects of PM_2.5_ exposure on CHD incidence may occur in the short term within 3 days.

### PM_2.5_ concentration in baseline and future periods

**Figure 2:**
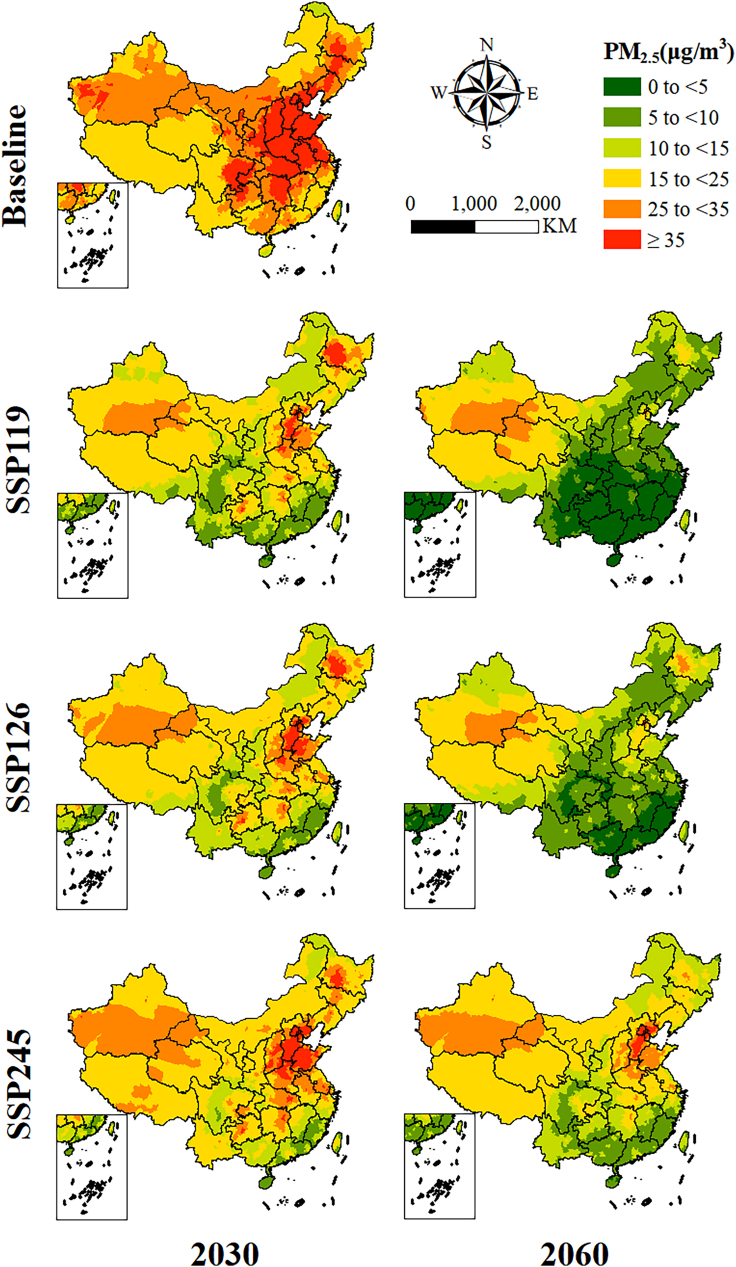
Spatial distribution of annual average PM_2.5_ concentration at baseline and in 2030 and 2060 under different SSP scenarios

Figure 2 showed that in the baseline years (2015–2020), the annual average PM_2.5_ concentration in most areas of the country did not meet the WHO interim target-3 (IT-3) standard (15 μg/m^3^). With the exception of the Pearl River Delta (PRD), the major urban agglomerations did not meet the IT-1 standard (35 μg/m^3^). Under all the three SSP scenarios, the PM_2.5_ concentration in China could show a downward trend in the future, and the areas with relatively high concentrations would still present in the Beijing-Tianjin-Hebei (BTH) region and parts of the northeastern regions. Under the most ideal SSP119 scenario (approximately carbon-neutral scenario), the average PM_2.5_ in China in 2060 could drop by 81.07% compared to the baseline. Under this scenario, most counties in the southern regions can meet the AQG standard recommended by the WHO, and other regions would also be under better control. Under the SSP126 scenario (approximately carbon-peak scenario), the overall PM_2.5_ concentration in the country could be close to but slightly higher than that under the SSP119 scenario, and the PM_2.5_ concentration could decrease by 74.48%. Regarding the SSP245 scenario, although the average PM_2.5_ concentration in 2060 would also drop by 53.17% compared to the baseline, there could still a large gap when referring to the concentration levels under the SSP119 and SSP126 scenarios (see Tables S3 and S4; Figure S3).

### Short-term PM_2.5_-related excess incidence of CHD and stroke

**Figure 3:**
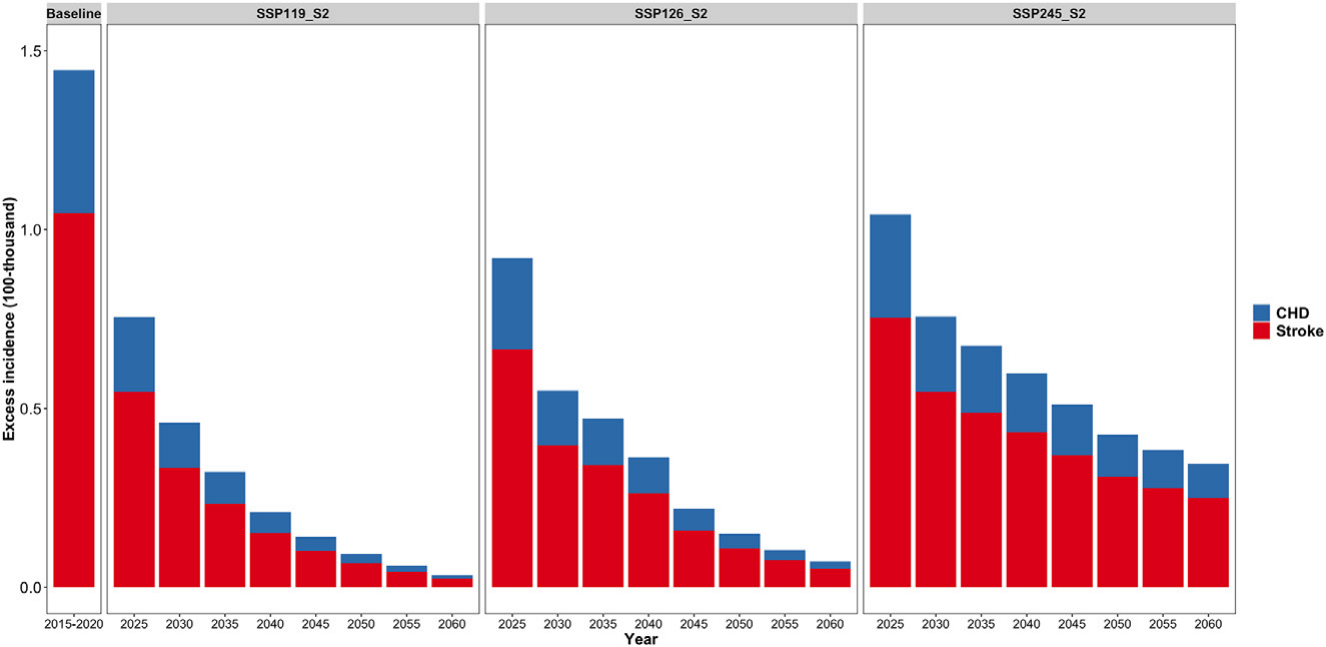
Annual average excess incidence related to short-term PM_2.5_ exposure at baseline and in future years under the combined scenarios of SSP2-S2 population and three SSPs

**Figure 4:**
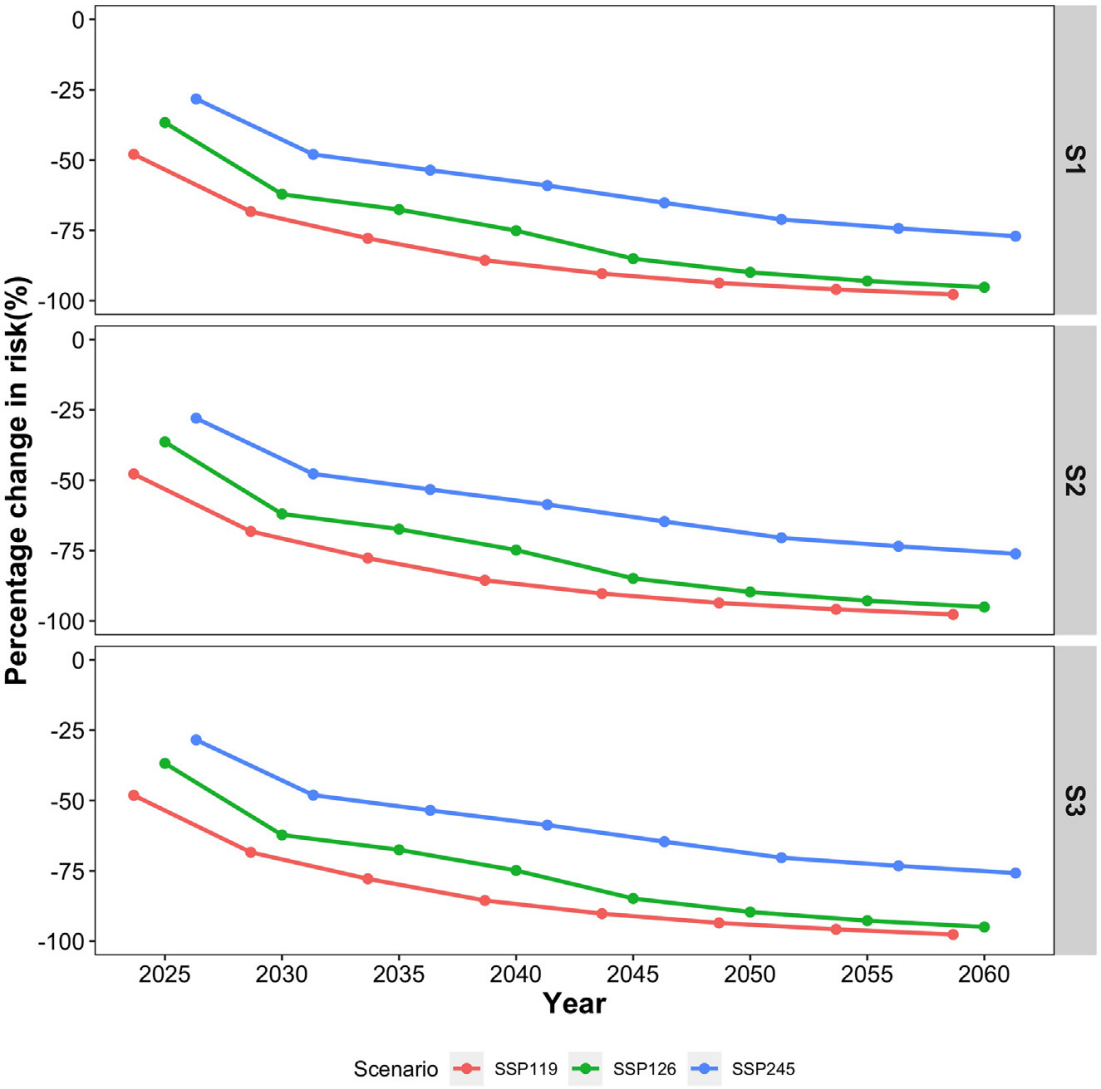
Changes in the excess incidence related to short-term PM_2.5_ exposure in future years under the combined scenarios of SSP2 (S1, S2, S3) population and three SSPs, as compared to the baseline

**Figure 5:**
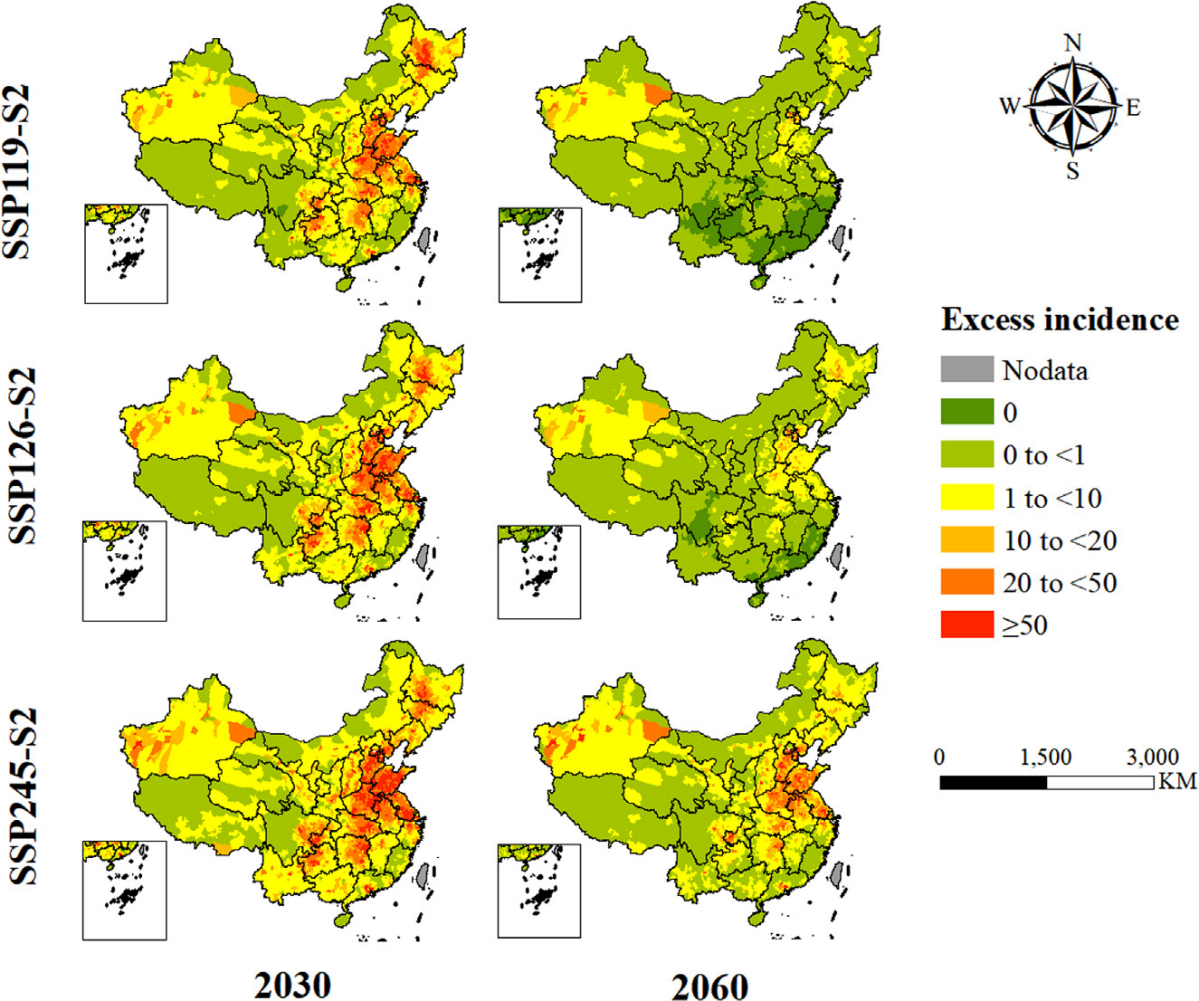
Spatial distribution of the excess incidence related to short-term PM_2.5_ exposure at baseline and in 2030 and 2060 under the combined scenarios of SSP2-S2 population and three SSPs

The annual mean excess incidence related to short-term PM_2.5_ exposure was 144,672 (95% CI: 40,532, 247,607) during the baseline of 2015–2020, including 104,603 (95% CI: 21,642, 186,554) stroke and 40,068 (95% CI: 18,890, 61,052) CHD. With the dual effects of future changes in the PM_2.5_ concentration and population in China, the excess risk of CHD and stroke associated with short-term PM_2.5_ exposure would gradually decrease over time (Table S5). Taking the SSP2-S2 population scenario as an example, the total excess incidence of the two diseases in 2060 would be reduced to 3,352 (95% CI: 939, 5,738) and 7,188 (95% CI: 2,013, 12,301) under the SSP119 and SSP126 scenarios, respectively, far less than 34,485 (95% CI: 9,662, 59,022) under the SSP245 scenario (see Figures 3 and 4). The corresponding additional economic burdens for CHD and stroke would be reduced to 13,548.8 thousand RMB yuan (95% CI: 6,394.8, 20,659) and 73,204.8 thousand RMB yuan (95% CI: 15,130.2, 130,554.6) in SSP119, respectively, which indicated a 95.03%–97.67% decrease compared to the baseline economic burden (Table S6). The same trend was observed among five different regions. Especially for the PRD region, the total excess incidence was projected to be close to zero around the year 2045 under SSP119 (Figure S4). Figure 5 shows the spatial distribution of short-term PM_2.5_-related excess incidence, which indicates that the BTH region, the most densely populated and heavily polluted region, could experience a dramatic reduction of its future excess incidence under the SSP119 and SSP126 scenarios.

### Contributors to the short-term PM_2.5_-related excess incidence

**Figure 6:**
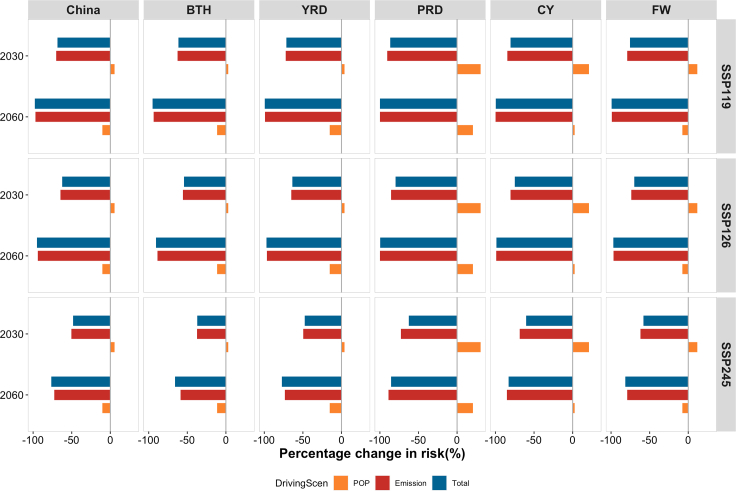
Contribution rate of emission and population in changes of excess incidence using medium population under SSP scenarios

Emissions and population were the two major contributors to the excess incidence, and our study found that emissions were the dominant factor. Figure 6 showed that in 2030, under all scenarios across each region, population could lead to the excess incidence increasing slightly, but the overall incidence burden could remain on a downward trend due to the large decline in PM_2.5_ concentrations. By 2060, population would still increase the excess incidence but only in the PRD and the Chengdu-Chongqing (CY) regions, while population changes in the BTH, the Yangtze River Delta (YRD), and the Fenwei Plain (FW) regions would cause a small decrease. Among them, the contributions from population changes in BTH and YRD were expected to be slightly lower than those of the other regions.

## Discussion

There is a need to establish a baseline relationship between cardiovascular disease and short-term exposure to PM_2.5_ and to project potential changes under future warming scenarios.

We found significant associations between short-term exposure to PM_2.5_ and the incidence risk of CHD and stroke, which is consistent with previous studies on the Chinese population. A 184-city time-series study reported estimated increases in the hospitalization risk associated with daily PM_2.5_ exposure of 0.31% and 0.29% for CHD and ischemic stroke, respectively^32^; the results of a 172-city study based on a basic medical insurance dataset reported that the risk of hospitalization for ischemic stroke increased by 0.34% (95% CI: 0.20%, 0.48%).^33^ Our estimates for the associated incidence risk of CHD and stroke were 0.83% and 0.73%, respectively, which are both higher than the results of previous national multicenter studies. Compared to the results of a meta-analysis, the estimation of the stroke incidence risk in this study is close to the pooled result of 1.1% (95% CI: 1.0%, 1.2%),^34^ reflecting the consistency with the results of international studies. The application of population-representative incidence data is critical for increasing the accuracy and power of the statistical results of this study.

The short-term PM_2.5_-associated excess incidence was projected to decrease in the future, especially under the approximately carbon-neutral scenario. There could be a maximum decline in the total incidence of cardiovascular diseases and related healthcare expenditures under SSP119 and a moderate decline under approximately carbon-peak scenario SSP126 and a minimum decline under current-situation scenario SSP245, implying that China’s carbon-neutral policies may gain higher health as well as economic benefits. This finding is generally consistent with previous studies despite differences in the projected risk type, scenarios, regions, exposure data used, and population.^16^^,^^19^^,^^35^^,^^36^ As reported, there were 47,000 avoided excess deaths in 74 Chinese cities in 2017 compared to 2013,^35^ while the attributable chronic mortality could be reduced by 370,000.^36^ The mitigation of greenhouse gases and pollution emission sources plays critical roles in bringing the benefits of eliminating climate change and air pollution, as well as subsequent health risk avoidance. Therefore, this study provided health-benefit-oriented policy support for China’s two-carbon target. In addition, we found that stoke was projected to remain the major cardiovascular disease with a higher excess incidence than CHD, which was consistent with previous evidence and thereby revealed a necessity to pay more attention to cardiovascular diseases sensitive to PM_2.5_ pollution.^37^

While the majority of regions in China are projected to experience a significant reduction in PM_2.5_ concentration from 2030 to 2060 under the approximately carbon-neutral scenario, the regions most affected by PM_2.5_ will persistently include the BTH, the Northeast Plain, and areas around Xinjiang Province in western China. The spatial pattern of excess incidence burden was close to the distribution of PM_2.5_ concentration under the same scenarios (see Figure 2) but with a larger extent due to the expansion of future populations (refer to Figure 5). On one hand, PM_2.5_ pollution in these regions could still exceed the WHO AQG levels under SSP119. According to the previous study, although there is a predicted reduction in PM_2.5_ concentration ranging from 6.3–11.1 μg/m^3^ compared to the baseline in a low-emission scenario (SSP126), the PM_2.5_ concentration may persist above the WHO limits of 25 μg/m^3^ until the year of 2050.^37^ On the other hand, the rapid growth of the regional population under PM_2.5_ exposure may offset the health benefits derived from PM_2.5_ emission reduction.^38^ Therefore, this finding reveals the necessity for continued and targeted emission control measures in these regions.^30^^,^^39^

Future excess incidence of cardiovascular diseases related to PM_2.5_ could be driven by the dual impacts from changes in emissions and population. According to the results of contribution to the change of future excess incidence nationwide and in five regions, we observed that emission reduction could play a critical role in health burden reduction, which was consistent with previous studies.^35^^,^^37^ For the entire country, the impact from population growth may contribute to increase the short-term PM_2.5_-associated excess incidence in 2030 but drive the reduction of the excess incidence in 2060. However, population growth could persistently contribute to increase excess incidence over time in the PRD and CY regions. Our findings suggested that emission reduction could be the most effective way to reduce the short-term PM_2.5_-associated disease burden,^1^^,^^2^^,^^40^ while regional differences due to population growth may require region-specific policy implementation.

This study performed a comprehensive projection of short-term PM_2.5_-associated excess incidence of cardiovascular diseases by using future scenarios aligned with carbon-neutral policies. Our results contribute to a better understanding of the health benefits associated with China’s carbon-neutral target. Meanwhile, this study also overcame the lack of exposure-response relationship between short-term exposure to PM_2.5_ and incidence of cardiovascular diseases, which provided another important term to evaluate PM_2.5_-associated health burdens other than the mortality. The results of this study should be considered in light of the following limitations and uncertainties. First, the health data on cardiovascular diseases used in this study do not cover the whole country due to data limitations. This may introduce uncertainty when estimating the excess incidence.^41^ Second, possible changes in the age structure are not considered, which may underestimate the excess incidence related to PM_2.5_. Third, incidence rates of CHD and stroke remained unchanged in the future projection due to data unavailability, which could bring uncertainties in the estimation of excess incidence.

In summary, China’s carbon-neutral policies may gain dramatic health benefits of a decreased burden of short-term PM_2.5_-associated cardiovascular diseases. The implementation of the carbon-neutral target could achieve air pollution control and health benefits. Since the regions that have been densely polluted in the past will still face a higher short-term PM_2.5_-associated cardiovascular disease burden in the future, targeted policy implementation should adapt to local conditions.

## Experimental procedures

### Resource availability

#### Lead contact

Further information and requests for resources should be directed to and will be fulfilled by the lead contact, Dr. Tiantian Li (litiantian@nieh.chinacdc.cn).

#### Materials availability

This study did not generate new unique materials.

#### Data and code availability

Daily PM_2.5_ concentration simulation database: https://github.com/KailaiLu/PM2.5_incidence_NC. Population database: https://springernature.figshare.com/collections/Provincial_and_gridded_population_projection_for_China_under_shared_socioeconomic_pathways_from_2010_to_2100/4605713. All analyses reported in this study used the statistical software R (v.4.2.2). The code used for the main analysis is available at https://github.com/KailaiLu/PM2.5_incidence_NC. Any additional information required for reanalyzing the data reported in this paper is available from the lead contact upon reasonable request.

### Association between short-term PM_2.5_ and incidence

We collected county-level individual incidence cases from 32 Chinese counties in 14 provinces between 2013 and 2018 from the Chinese Environmental Public Health Tracking system.^42^ The International Classification of Diseases, Tenth Revision (ICD-10), was adopted to identify two causes: CHD (I20-I25) and stroke (I60-I64).

We conducted a time-stratified case-crossover study to obtain the associations between CHD or stroke incidence and short-term exposure to PM_2.5_. In this study, the case days were the dates of disease incidence; the control days were restricted to days falling on the same day of the week, month, and year as the case days to control for time-varying and time-invariant individual confounders. Using this time-stratified approach, each case day could be matched with 3–4 control days. We then fitted a conditional logistic regression model to obtain the exposure-response relationship between PM_2.5_ and incidence of CHD or stroke.^43^ The regression model incorporated county-level daily average PM_2.5_ as a main effect and controlled several covariates including (1) a smoothing function using natural splines (ns) with three degrees of freedom (df) for both the daily temperature and the relative humidity in the model and (2) a dichotomous variable for adjusting the holiday effects (1 = holiday; 0 = non-holiday). To investigate the delayed effect of PM_2.5_, we separately fitted the models considering different lags up to 3 days prior to the date of incidence, including single-day lags (lag1, lag2, lag3) and cumulative-day lags (lag0-1, lag0-2, lag0-3). We reported the odds ratio (OR) and 95% CI to indicate the association between daily PM_2.5_ and the incidence of CHD and stroke.

### Predicting future PM_2.5_ concentrations

We applied China’s future anthropogenic emission datasets from the dynamic projection model for emissions in China (DPEC)^7^ to simulate future PM_2.5_ concentrations in this work. The DPEC model contains a series of Chinese emission pathways from 2015 to 2060 under a range of socioeconomic, climate, and pollution control policies,^7^ and three scenarios, namely SSP119, SSP126, and SSP245, were selected to represent the impacts of China’s carbon-neutrality policy, carbon-peak policy, and current situation, respectively.

We then utilized the offline coupled Weather Research and Forecasting (WRF; v.3.9.1)-Community Multiscale Air Quality (CMAQ; v.5.2) air quality modeling system to obtain the variations in future PM_2.5_ concentrations by 2060 under the three scenarios above. The air quality modeling covered mainland China with a spatial resolution of 36 km and a vertical resolution of 14 layers. Each scenario was simulated for the whole year with a 1-month spin up. Meteorological inputs were provided by WRF simulations and were fixed to the base-year (i.e., 2015) level. For emission inputs, China’s historical and future anthropogenic emissions were derived from the MEIC^44^ and DPEC^45^ models, respectively; data for neighboring countries were obtained from the MIX^46^ and CMIP6^47^ databases, respectively. Natural source emissions, including biogenic (from MEGANv.2.1^48^), open biomass burning (from the GFED4 database^49^), and dust emissions (online simulation) were all included. The initial and boundary chemical conditions were dynamically provided by GEOS-Chem simulations. Detailed above-mentioned configurations, as well as meteorology parameterizations and chemical mechanism, are documented in Table S1.

Furthermore, to reduce the uncertainty introduced by the chemical transport model, we used observation-based PM_2.5_ hindcast datasets to calibrate the daily WRF-CAMQ simulation by grid as follows:

(Equation 1) $$C_{i,j}={C_{TAP}}_{2015}\times \frac{{C_{SIM}}_{i,j}}{{C_{SIM}}_{base,2015}}$$

where $C_{i,j}$ represents the predicted PM_2.5_ concentration of scenario i in year j. ${C_{TAP}}_{2015}$ is the base-year (i.e., 2015) PM_2.5_ hindcast concentration. ${C_{SIM}}_{i,j}$ and ${C_{SIM}}_{base,2015}$ represent the WRF-CMAQ-simulated PM_2.5_ concentrations of scenario i in year j and of the base year, respectively.

### Incidence rate and population projection

The nation-level baseline incidence rates of CHD and stroke were calculated by dividing the sum of the annual average cases by the total census population of the 32 counties from 2013 to 2018. We assumed that the baseline rate would remain stable in the future. We matched the population data for the year corresponding to the PM_2.5_ exposure data. We applied three scenarios under the SSP2 population scenario, including S1 (low fertility rate), S2 (medium fertility rate), and S3 (high fertility rate). The gridded SSP2 population at a 1 × 1 km resolution was from previous work^50^ in which the future yearly Chinese population was extracted and reanalyzed based on a global population projection by considering recent fertility-promoting policies implemented in China. The gridded population was calculated at the county level by summing the population in each grid within the county.

### Projection of short-term PM_2.5_-associated excess incidence

Based on the data presented above, we computed the daily excess incidence related to short-term PM_2.5_ exposure for each county as follows:

(Equation 2) $${\Delta Incidence}_{i}=\sum_{j=1,\ldots ,n}{POP}_{i}\times Y\times AF\times \left(C_{ij}-C_{0}\right)$$

where *ΔIncidence*_*i*_ represents the daily excess incidence associated to short-term PM_2.5_ exposure for county *i* on day *j* during each year; *POP*_*i*_ is the total population of county *i* in the corresponding year; *Y* is the incidence rate of each disease; *AF* is the attributable fraction of stroke or CHD incidence associated with short-term PM_2.5_ exposure, which was calculated using (OR-1)/OR derived from the results of the exposure-response relationship analysis (we assumed that the *AF* was nationally consistent and stable in the future); *C*_*ij*_ is the county-level daily average PM_2.5_ concentration in county *i* on day *j*; and *C*_0_ is the AQG level of daily average PM_2.5_ concentration set at 15 μg/m^3^, which is an evidence-informed derivation of the AQG level recommended by the WHO in 2021. Therefore, the associated excess incidence can only be calculated when *C*_*ij*_ exceeds *C*_*0*_. We first calculated the daily level, county-specific PM_2.5_-associated excess incidence of stroke and CHD across the country from 2015 to 2020 as the baseline, and we projected the excess incidence from 2025 up to 2060 at 5-year intervals (namely 2025, 2030, 2035, 2040, 2045, 2050, 2055, and 2060) under the combined scenarios of three PM_2.5_ emission control scenarios (SSP119, SSP126, and SSP245) and three SSP2 population scenarios (S1, S2, and S3) to predict the diversity of future changes. Following this, we computed the annual average excess incidence related to short-term PM_2.5_ in each county for the baseline and for each projection year. Subsequently, we aggregated the annual excess incidence number in each county according to different geographical extents including the entire country and five city agglomerations including the BTH, the YRD, the PRD, the FW, and the CY regions.

Moreover, to evaluate the potential economic burden related to the excess incidence of CHD and stroke, we computed the annual mean additional expenditure for both baseline and future years across various scenarios. We utilized the average per-hospitalization costs for CHD (14,600 RMB yuan) and stroke (30,200 RMB yuan), published in the Report on Cardiovascular Health and Diseases in China, serving as a proxy for the overall expenditure of disease treating.^51^ Then, we calculated the additional expenditure by combining the excess incidence numbers with the individual costs for CHD and stroke, respectively. Following that, we compared the annual mean additional expenditure in future years across various scenarios to that of baseline to quantify the economic benefits.

To explore the contribution to future incidence changes from the impact of population and emission control, we set up three virtual scenarios. During this phase, we set a new baseline, which engaged population in 2020 and daily average PM_2.5_ concentration from 2015 to 2020. The results of this new baseline may be very close to the previous baseline, but to calculate the impact of the two factors more accurately, we use this baseline to exclude errors caused by different calculation methods, and the new baseline was used only for calculating the contribution of each driving factor. Then, we set up the population scenario (POP) and the emission scenario (emission). The POP scenario used the same PM_2.5_ concentration data as the new baseline scenario and the SSP2-S2 future population, while the emission scenario used the 2020 population, consistent with the new baseline scenario with future PM_2.5_ concentrations under SSP119, SSP126, and SSP245. The results could represent the respective contributions from the changes in PM_2.5_ exposure and population. Using the same method, we also conducted regional analysis on the five key regions (BTH, YRD, PRD, CY, and FW) in China.

All analyses were conducted in R 4.0.3 (R Foundation for Statistical Computing, Vienna, Austria) and ArcGIS 10.2 (ESRI, Redlands, CA, USA).

## Supplemental information

Document S1. Figures S1–S4 and Tables S1–S6

Document S2. Article plus supplemental information
