# Supplementary material for: China’s carbon-neutral policies will reduce short-term PM2.5-associated excess incidence of cardiovascular diseases
Source: One Earth. 2024 Mar 15;7(3):497–505. doi: 10.1016/j.oneear.2024.01.006 (PMC10962059; doi:10.1016/j.oneear.2024.01.006)
Supplement: Document S1. Figures S1–S4 and Tables S1–S6 [file mmc1.pdf]

One Earth, Volume 7

Supplemental information

China's carbon-neutral policies will reduce short-term $PM_{2.5}$-associated excess incidence of cardiovascular diseases

Jie Ban, Jing Cheng, Can Zhang, Kailai Lu, Zhen Zhou, Zhao Liu, Yidan Chen, Can Wang, Wenjia Cai, Peng Gong, Yong Luo, Dan Tong, Jianlin Hu, Xinbiao Guo, Junwei Hao, and Tiantian Li

Supplementary

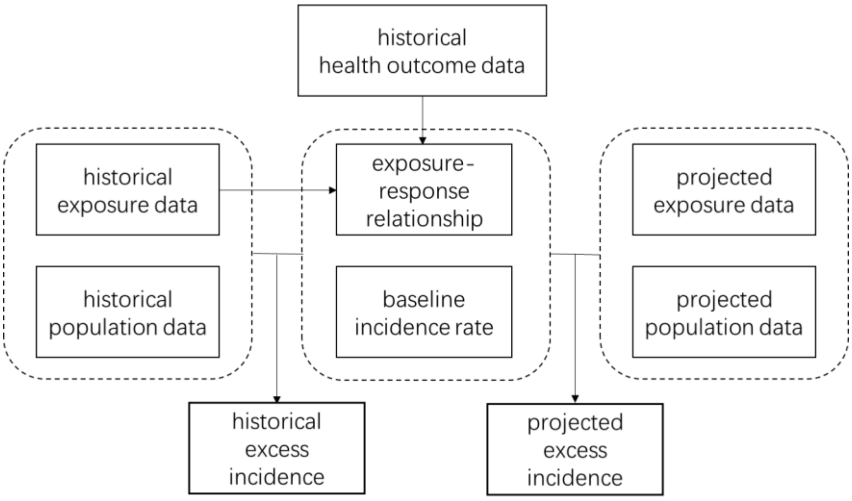

Figure S1. Framework of integrated analysis process

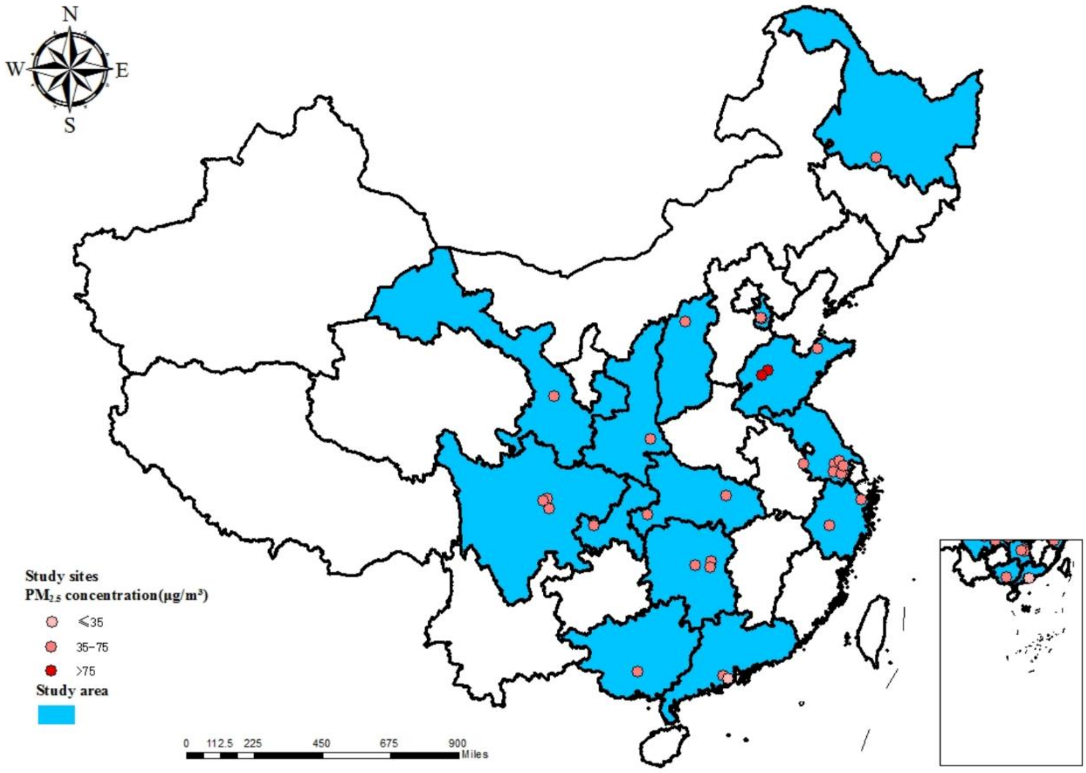

Figure S2. Map of studied counties

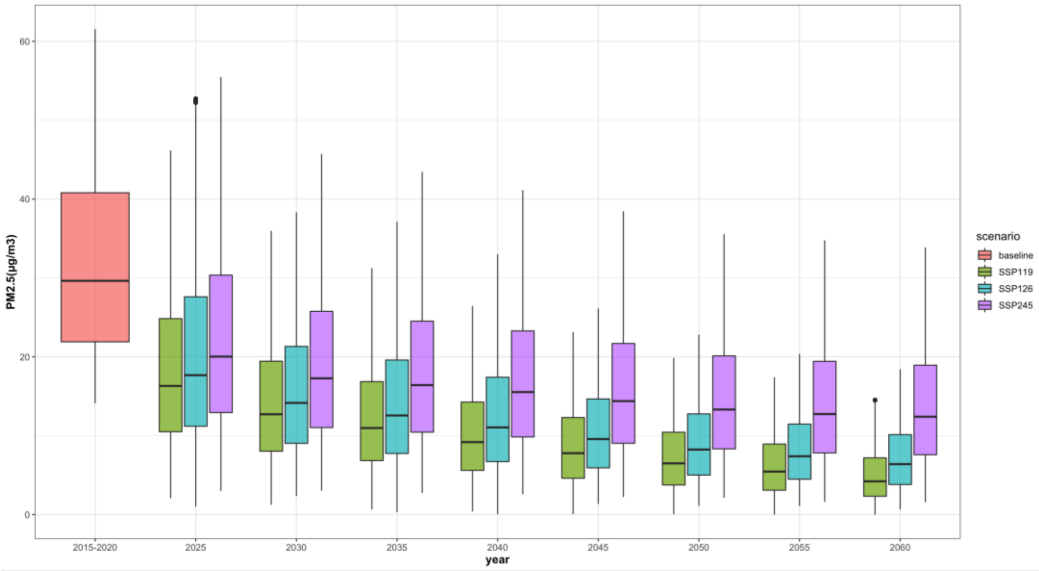

Figure S3. Distribution of county-level annual mean $PM_{2.5}$ concentration under different emission scenarios in each year. Error bars represent the minimum and maximum concentration values.

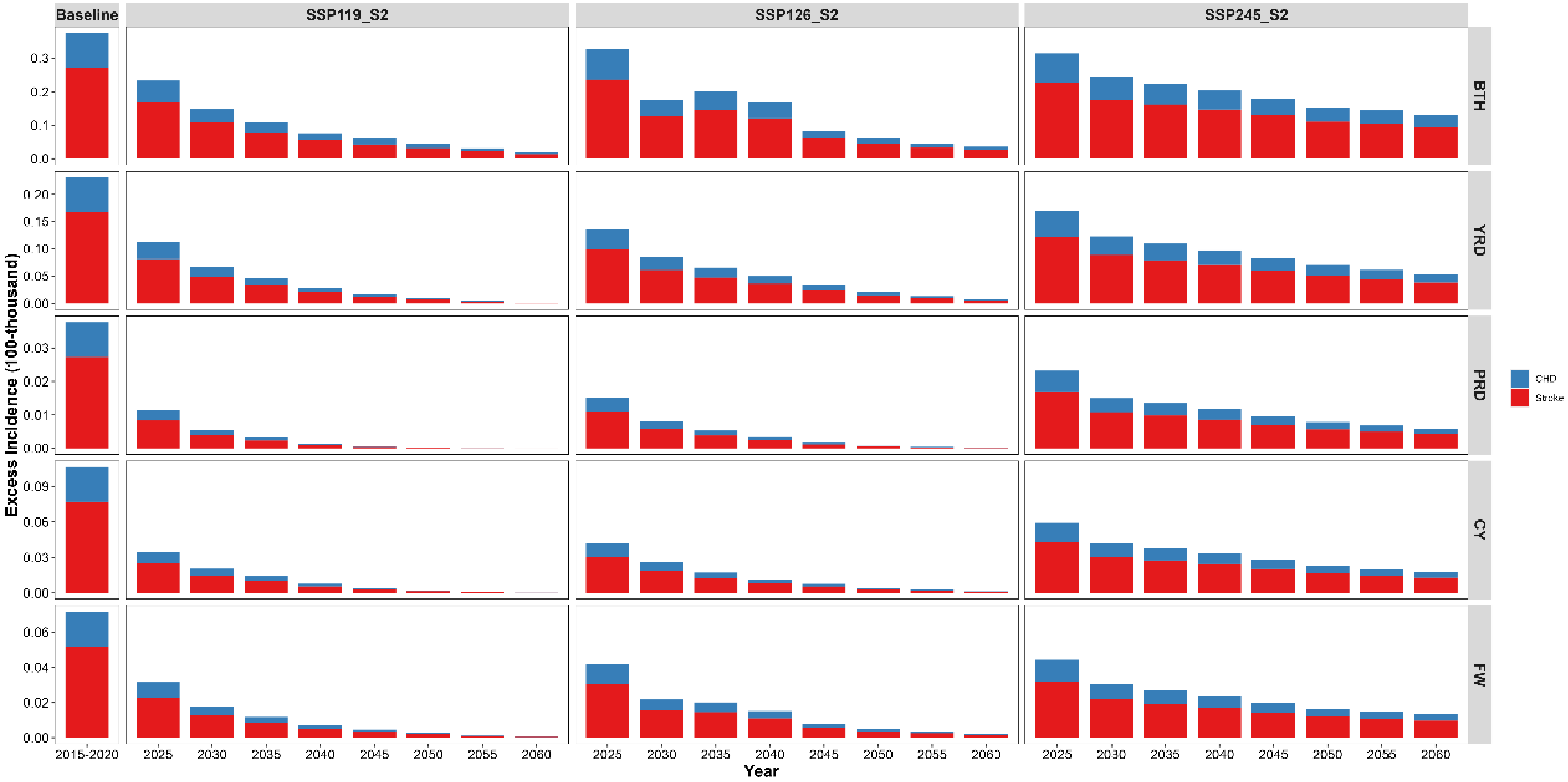

Figure S4. Annual mean excess incidence of CHD and stroke in each region under the combined scenarios of SSP2-S2 population and three SSPs

Table S1. WRF-CMAQ modelling system configurations

Model	Parameter	Configuration schemes
WRF	Horizontal resolution	36km, 181 (column) × 136 (row)
	Vertical resolution	28 sigma levels from surface to tropopause
	Simulation period	The whole year with one-month spin-up (All CMAQ simulations applied the WRF meteorology fixed in 2015)
	IC and BC	Final analysis data from NCEP
	Shortwave radiation	New Goddard scheme
	Longwave radiation	RRTM scheme
	Surface layer	Pleim–Xiu scheme
	Planetary boundary layer	ACM2 scheme
	Cumulus	Kain–Fritsch scheme
	Cloud microphysics	WSM6 scheme
CMAQ	Horizontal resolution	36km, 172 (column) × 127 (row)
	Vertical resolution	14 sigma levels from surface to tropopause
	Simulation period	The whole year with one-month spin-up
	IC and BC	Dynamic GEOS-Chem global simulation outputs
	Gas-phase mechanism	CB05
	Aqueous-phase mechanism	RADM
	Aerosol module	AERO6
	Cloud module	ACM_AE6 ACM cloud processor
	Photolytic rate	In-line calculation
	Anthropogenic emissions	Historical: MEIC for China; MIX for other Asian countries<br>Future: DPEC for China; CMIP6 for other Asian countries
	Biogenic emissions	MEGANv2.1
	Open biomass burning	GFED4
	Dust	In-line calculation
	Lightning	Not included

Table S2. Summary description on environmental exposure

	Mean	Min	P25	P75	Max
$PM_{2.5}$ (μg/m^3)	53.4	3.0	26.5	67.2	647.8
O_3-8h (μg/m^3)	89.5	1.0	53.6	117.6	637.0
Temperature (℃)	16.0	-31.5	8.8	23.9	35.8
Relative humidity (%)	71.0	11.3	62.0	83.0	100.0

Table S3. Statistics of $PM_{2.5}$ concentration

Scenario	Year	Mean(μg/m^3)	Stdev	Min	P50	P75	Max
Baseline	2015-2020	37.91	16.08	1.01	34.46	48.41	85.54
SSP119	2025	24.16	11.75	0.73	21.16	30.90	82.78
	2030	18.65	9.04	0.60	16.67	23.61	73.12
	2035	15.99	7.91	0.53	14.33	20.05	72.44
	2040	13.45	6.78	0.45	12.24	16.77	62.64
	2045	11.63	6.17	0.39	10.56	14.65	54.40
	2050	9.98	5.42	0.34	9.05	12.90	40.71
	2055	8.68	5.00	0.30	7.80	11.23	33.47
	2060	7.18	4.55	0.26	6.27	9.29	31.06
SSP126	2025	26.93	14.19	0.81	23.53	33.25	99.37
	2030	20.39	9.35	0.66	18.33	25.85	60.93
	2035	18.74	10.07	0.60	16.57	22.97	78.26
	2040	16.57	9.05	0.54	14.78	20.27	71.27
	2045	13.78	6.39	0.46	12.67	17.46	47.69
	2050	11.98	5.62	0.40	11.10	15.22	41.24
	2055	10.76	5.02	0.36	9.97	13.54	35.92
	2060	9.56	4.82	0.31	8.79	12.18	33.50
SSP245	2025	29.29	13.69	0.91	26.33	37.23	96.30
	2030	24.43	11.03	0.79	22.39	30.48	80.27
	2035	23.15	10.52	0.74	21.25	28.78	81.55
	2040	21.89	9.98	0.70	20.09	27.22	79.10
	2045	20.38	9.41	0.66	18.81	25.49	75.12
	2050	18.87	8.70	0.62	17.51	23.78	68.12
	2055	18.23	8.76	0.59	16.86	22.93	69.33
	2060	17.75	8.50	0.57	16.52	22.32	67.10

Table S4. Change of concentration under difference scenarios

Year	SSP119	SSP126	SSP245
2025	-13.74(-36.26%)	-10.98(-28.96%)	-8.61(-22.72%)
2030	-19.26(-50.81%)	-17.52(-46.22%)	-13.48(-35.56%)
2035	-21.92(-57.83%)	-19.16(-50.55%)	-14.76(-38.94%)
2040	-24.46(-64.53%)	-21.34(-56.29%)	-16.02(-42.25%)
2045	-26.28(-69.33%)	-24.13(-63.65%)	-17.52(-46.23%)
2050	-27.92(-73.66%)	-25.92(-68.39%)	-19.03(-50.21%)
2055	-29.23(-77.10%)	-27.15(-71.62%)	-19.68(-51.92%)
2060	-30.73(-81.07%)	-28.35(-74.78%)	-20.15(-53.17%)

Table S5. The excess incidence of stroke and CHD under difference combined scenarios of population and three SSPs

Emission scenarios	Year	Stroke			CHD		
		SSP2-S1	SSP2-S2	SSP2-S3	SSP2-S1	SSP2-S2	SSP2-S3
SSP119	2025	54428 (11261, 97070)	54653 (11308, 97471)	54242 (11222, 96738)	20849 (9829, 31767)	20935 (9870, 31899)	20777 (9795, 31659)
	2030	33148 (6858, 59119)	33288 (6887, 59368)	33042 (6836, 58929)	12698 (5986, 19347)	12751 (6011, 19429)	12657 (5967, 19285)
	2035	23198 (4800, 41372)	23322 (4825, 41594)	23179 (4796, 41339)	8886 (4189, 13540)	8934 (4212, 13612)	8879 (4186, 13529)
	2040	15041 (3112, 26825)	15163 (3137, 27042)	15111 (3126, 26949)	5762 (2716, 8779)	5808 (2738, 8850)	5788 (2729, 8819)
	2045	10073 (2084, 17965)	10205 (2111, 18200)	10214 (2113, 18216)	3859 (1819, 5879)	3909 (1843, 5956)	3913 (1845, 5962)
	2050	6588 (1363, 11749)	6725 (1391, 11993)	6765 (1400, 12064)	2524 (1190, 3845)	2576 (1214, 3925)	2591 (1222, 3948)
	2055	4219 (873, 7525)	4346 (899, 7751)	4397 (910, 7842)	1616 (762, 2463)	1665 (785, 2537)	1684 (794, 2566)
	2060	2329 (482, 4154)	2424 (501, 4323)	2470 (511, 4406)	892 (421, 1359)	928 (438, 1415)	946 (446, 1442)
SSP126	2025	66266 (13710, 118182)	66567 (13772, 118719)	66099 (13676, 117884)	25383 (11967, 38676)	25499 (12021, 38852)	25319 (11937, 38579)
	2030	39592 (8192, 70611)	39770 (8228, 70927)	39482 (8169, 70413)	15166 (7150, 23108)	15234 (7182, 23212)	15124 (7130, 23044)
	2035	33901 (7014, 60460)	34130 (7061, 60869)	33964 (7027, 60572)	12986 (6122, 19786)	13074 (6163, 19920)	13010 (6133, 19823)
	2040	26072 (5394, 46497)	26329 (5447, 46957)	26277 (5437, 46865)	9987 (4708, 15217)	10086 (4755, 15367)	10066 (4745, 15337)
	2045	15645 (3237, 27902)	15854 (3280, 28275)	15860 (3281, 28285)	5993 (2825, 9131)	6073 (2863, 9253)	6075 (2864, 9257)
	2050	10566 (2186, 18845)	10783 (2231, 19232)	10832 (2241, 19318)	4047 (1908, 6167)	4131 (1947, 6294)	4149 (1956, 6322)
	2055	7324 (1515, 13062)	7540 (1560, 13448)	7610 (1575, 13572)	2806 (1323, 4275)	2888 (1362, 4401)	2915 (1374, 4442)
	2060	5006 (1036, 8928)	5197 (1075, 9268)	5277 (1092, 9411)	1918 (904, 2922)	1991 (938, 3033)	2021 (953, 3080)
SSP245	2025	75047 (15527, 133843)	75384 (15597, 134444)	74851 (15486, 133494)	28747 (13553, 43802)	28876 (13614, 43998)	28672 (13517, 43687)
	2030	54402 (11256, 97023)	54674 (11312, 97508)	54314 (11237, 96866)	20839 (9824, 31752)	20943 (9873, 31911)	20805 (9808, 31700)
	2035	48547 (10044, 86582)	48874 (10112, 87164)	48630 (10061, 86729)	18596 (8767, 28335)	18721 (8826, 28525)	18628 (8782, 28383)
	2040	42852 (8866, 76425)	43271 (8953, 77171)	43173 (8932, 76998)	16415 (7739, 25011)	16575 (7814, 25255)	16538 (7797, 25198)
	2045	36412 (7534, 64939)	36943 (7643, 65885)	36998 (7655, 65984)	13948 (6576, 21252)	14151 (6671, 21562)	14172 (6681, 21594)

2050	30229 (6254, 53912)	30876 (6388, 55066)	31037 (6421, 55352)	11579 (5459, 17643)	11827 (5576, 18021)	11889 (5605, 18115)
2055	26919 (5570, 48009)	27728 (5737, 49451)	27993 (5792, 49925)	10312 (4861, 15712)	10621 (5007, 16183)	10723 (5055, 16338)
2060	23998 (4965, 42800)	24934 (5159, 44469)	25317 (5238, 45152)	9193 (4334, 14007)	9551 (4503, 14553)	9698 (4572, 14777)

Table S6. Annual mean additional hospitalization cost related to excess incidence of two diseases under the combined SSP2-S2 population and three SSPS

Scenario	Year	Additional hospitalization cost (thousand RMB)	
		CHD (95%CI)	Stroke (95%CI)
Baseline	2015-2020	584,992.8(275,794, 891,359.2)	3,159,011(653,588.4, 5,633,931)
SSP119	2025	305,651 (144,102, 465,725.4)	1,650,520.6 (341,501.6, 2,943,624.2)
	2030	186,164.6 (87,760.6, 283,663.4)	1,005,297.6 (207,987.4, 1,792,913.6)
	2035	130,436.4 (61,495.2, 198,735.2)	704,324.4 (145,715, 1,256,138.8)
	2040	84,796.8 (39,974.8, 129,210)	457,922.6 (94,737.4, 816,668.4)
	2045	57,071.4 (26,907.8, 86,957.6)	308,191 (63,752.2, 549,640)
	2050	37,609.6 (17,724.4, 57,305)	203,095 (42,008.2, 362,188.6)
	2055	24,309 (11,461, 37,040.2)	131,249.2 (27,149.8, 234,080.2)
	2060	13,548.8 (6,394.8, 20,659)	73,204.8 (15,130.2, 130,554.6)
SSP126	2025	372,285.4 (175,506.6, 567,239.2)	2,010,323.4 (415,914.4, 3,585,313.8)
	2030	222,416.4 (104,857.2, 338,895.2)	1,201,054 (248,485.6, 2,141,995.4)
	2035	190,880.4 (89,979.8, 290,832)	1,030,726 (213,242.2, 1,838,243.8)
	2040	147,255.6 (69,423, 224,358.2)	795,135.8 (164,499.4, 1,418,101.4)
	2045	88,665.8 (41,799.8, 135,093.8)	478,790.8 (99,056, 853,905)
	2050	60,312.6 (28,426.2, 91,892.4)	325,646.6 (67,376.2, 580,806.4)
	2055	42,164.8 (19,885.2, 64,254.6)	227,708 (47,112, 406,129.6)
	2060	29,068.6 (13,694.8, 44,281.8)	156.949.4 (32.465, 279.893.6)
SSP245	2025	421,589.6 (198,764.4, 642,370.8)	2.276.596.8 (471.029.4, 4.060.208.8)
	2030	305,767.8 (144,145.8, 465,900.6)	1.651.154.8 (341.622.4, 2.944.741.6)
	2035	273,326.6 (128,859.6, 416,465)	1.475.994.8 (305.382.4, 2632.352.8)

2040	241,995 (114,084.4, 368,723)	1,306,784.2 (270,380.6, 2,330,564.2)
2045	206,604.6 (97,396.6, 314,805.2)	1,115,678.6 (230,818.6, 1,989,727)
2050	172,674.2 (81,409.6, 263,106.6)	932,455.2 (192,917.6, 1,662,993.2)
2055	155,066.6 (73,102.2, 236,271.8)	837,385.6 (173,257.4, 1,493,420.2)
2060	139,444.6 (65,743.8, 212,473.8)	753,006.8 (155,801.8, 1,342,963.8)
