# Supplementary material for: China’s carbon-neutral policies will reduce short-term PM2.5-associated excess incidence of cardiovascular diseases
Source: One Earth. 2024 Mar 15;7(3):497–505. doi: 10.1016/j.oneear.2024.01.006 (PMC10962059; doi:10.1016/j.oneear.2024.01.006)
Supplement: Document S2. Article plus supplemental information [file mmc2.pdf]

One Earth

Article

# China's carbon-neutral policies will reduce short-term $PM_{2.5}$-associated excess incidence of cardiovascular diseases

## Graphical abstract

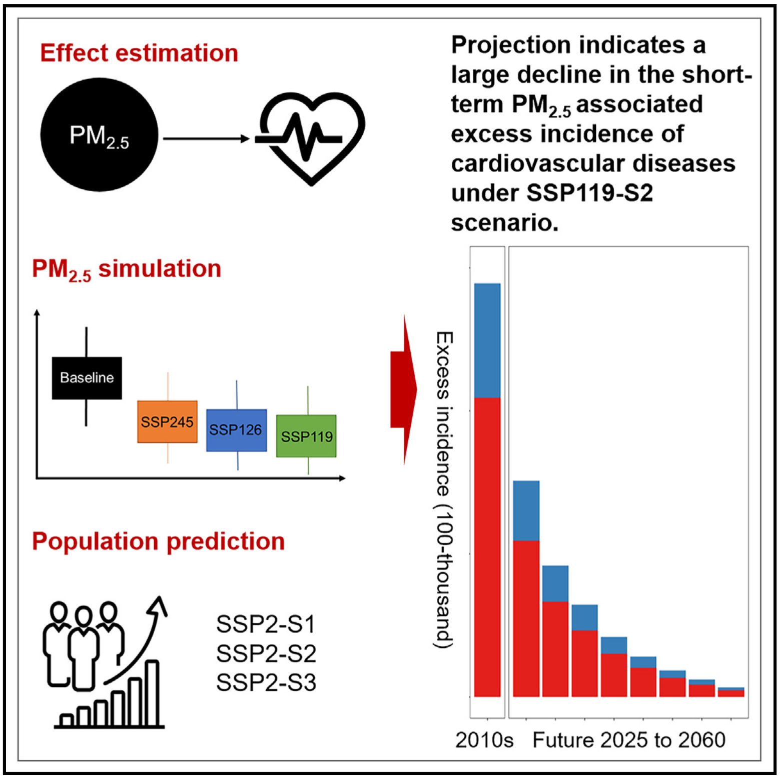


## Highlights

- $PM_{2.5}$ of SSP119 in 2060 will drop by 81.07% compared to the average level in 2015–2020
- Short-term $PM_{2.5}$-related excess incidence of cardiovascular diseases will be reduced to 3,352
- Emission control could be the key driving factor for the related health benefits

## Authors

Jie Ban, Jing Cheng, Can Zhang, ..., Xinbiao Guo, Junwei Hao, Tiantian Li

## Correspondence

litiantian@nieh.chinacdc.cn (T.L.), haojunwei@vip.163.com (J.H.)

## In brief

China's commitment to carbon neutrality may alter $PM_{2.5}$ pollution and its associated disease burdens. Projecting forward to 2060 under different carbon mitigation targets, this comprehensive projection study finds substantial reduction in annual average $PM_{2.5}$ levels and the short-term $PM_{2.5}$-associated excess incidence of cardiovascular diseases under SSP119 (approximately carbon-neutral scenario) in 2060 compared to the 2015–2020 baseline. Emission control is the dominant contributor driving these declines in different regions. China's carbon-neutral policies will bring health benefits and potential savings in healthcare expenditures.

Ban et al., 2024, One Earth 7, 497–505
March 15, 2024 © 2024 The Author(s). Published by Elsevier Inc.
https://doi.org/10.1016/j.oneear.2024.01.006

CellPress

One Earth

CellPress
OPEN ACCESS

Article

# China's carbon-neutral policies will reduce short-term $PM_{2.5}$-associated excess incidence of cardiovascular diseases

Jie Ban,[1,2,3,12] Jing Cheng,[4,12] Can Zhang,[1,2] Kailai Lu,[1,2] Zhen Zhou,[1,2] Zhao Liu,[5] Yidan Chen,[6] Can Wang,[6] Wenjia Cai,[4] Peng Gong,[7] Yong Luo,[4] Dan Tong,[4] Jianlin Hu,[3] Xinbiao Guo,[8] Junwei Hao,[9,10,11,*] and Tiantian Li[1,2,13,*]

[1]National Key Laboratory of Intelligent Tracking and Forecasting for Infectious Diseases, National Institute of Environmental Health, Chinese Center for Disease Control and Prevention, Beijing 100021, China
[2]China CDC Key Laboratory of Environment and Population Health, National Institute of Environmental Health, Chinese Center for Disease Control and Prevention, Beijing 100021, China
[3]Collaborative Innovation Center of Atmospheric Environment and Equipment Technology, Jiangsu Key Laboratory of Atmospheric Environment Monitoring and Pollution Control (AEMPC), Nanjing University of Information Science & Technology, Nanjing 210044, China
[4]Department of Earth System Science, Ministry of Education Key Laboratory for Earth System Modeling, Institute for Global Change Studies, Tsinghua University, Beijing 100084, China
[5]School of Linkong Economics and Management, Beijing Institute of Economics and Management, Beijing 100024, China
[6]State Key Joint Laboratory of Environment Simulation and Pollution Control (SKLESPC), School of Environment, Tsinghua University, Beijing 100084, China
[7]Department of Earth Sciences and Geography, University of Hong Kong, Hong Kong Special Administrative Region 999077, China
[8]Department of Occupational and Environmental Health Sciences, School of Public Health, Peking University, No. 38 Xueyuan Road, Beijing 100191, China
[9]Department of Neurology, Xuanwu Hospital, Capital Medical University, National Center for Neurological Disorders, No. 45 Changchun Street, Xicheng District, Beijing 100053, China
[10]Key Laboratory for Neurodegenerative Diseases of Ministry of Education, No. 45 Changchun Street, Xicheng District, Beijing 100053, China
[11]Beijing Municipal Geriatric Medical Research Center, No. 45 Changchun Street, Xicheng District, Beijing 100053, China
[12]These authors contributed equally
[13]Lead contact
*Correspondence: litiantian@nieh.chinacdc.cn (T.L.), haojunwei@vip.163.com (J.H.)
https://doi.org/10.1016/j.oneear.2024.01.006

**SCIENCE FOR SOCIETY** Ambient fine particulate matter ($PM_{2.5}$) can penetrate deep into the cardiorespiratory system and trigger cardiovascular diseases. In 2019, ambient $PM_{2.5}$ caused almost 1 million premature cardiovascular deaths in China. China's commitment to attaining carbon neutrality by 2060 may help reduce this burden given the potential decrease in $PM_{2.5}$ as a consequence of carbon dioxide emissions control. However, the impact of China's carbon neutrality target on $PM_{2.5}$-associated incidence of cardiovascular diseases remains unclear due to a lack of sophisticated analysis. Projections generated in this study indicate that China's carbon neutrality policies could alleviate the short-term $PM_{2.5}$-associated excess incidence of cardiovascular disease by 90.3% in 2060 relative to policies in place between 2015 and 2020. The implementation of continuous, region-specific emission reduction strategies by policymakers is crucial to achieve effective co-control of local $PM_{2.5}$ pollution and associated public health benefits.

## SUMMARY

China's carbon-neutral target could have benefits for ambient fine particulate matter ($PM_{2.5}$)-associated mortality. Although previous studies have researched such benefits, the potential impact on cardiovascular disease incidence burden is yet to be investigated thoroughly. Here, we first estimate the association between short-term $PM_{2.5}$ exposure and the incidence of stroke and coronary heart disease (CHD) via a case-crossover study before projecting future changes in short-term $PM_{2.5}$-associated excess incidence across China from 2025 to 2060 under three different emission scenarios. We find that, compared to the 2015–2020 baseline, average $PM_{2.5}$ concentrations nationwide in 2060 under SSP119 (an approximation of a carbon-neutral scenario) are projected to decrease by 81.07%. The short-term $PM_{2.5}$-related excess incidence of stroke and CHD is projected to be reduced to 3,352 cases (95% confidence interval: 939, 5,738)—compared with 34,485


This is an open access article under the CC BY license (http://creativecommons.org/licenses/by/4.0/).

cases under a medium-emissions scenario (SSP245)—and is expected to be accompanied by a 95% reduction in the related economic burden. China's carbon-neutral policies are likely to bring health benefits for cardiovascular disease by reducing short-term $PM_{2.5}$-related incidence burden.

## INTRODUCTION

Ambient fine particulate matter ($PM_{2.5}$) has caused 1,423 thousand deaths in China, with over 60% attributable to cardiovascular deaths.[1–3] Although a series of clean air policies are reported to have resulted in considerable improvement in China's air quality,[4–9] stronger policies are still required to substantially reduce the concentration of $PM_{2.5}$ to protect public health in the country[10] and to align with the World Health Organization's (WHO) newly announced global air quality guidelines (AQG). In September 2020, a carbon-neutral target was launched by the Chinese government to mitigate the effects of climate change. This target includes the objective of peaking $CO_2$ emissions before 2030 and achieving carbon neutrality before 2060. Considering that the causes and solutions of air pollution and climate change are closely linked,[11] mitigating climate change may also bring air quality improvement and have potential benefits for the health risks associated with ambient $PM_{2.5}$.[7,12] One of the associated health risks, cardiovascular diseases, is rising in incidence in China[13–15] and thus requires particular attention. However, to assess the potential effectiveness of China's coordinated governance of climate change and air pollution, we must first determine the incidence of cardiovascular diseases associated with ambient $PM_{2.5}$ and project how these might change in the future.

Previous studies have projected the future $PM_{2.5}$-associated mortality under different scenarios of climate control and air pollution policies in China[7,16–21] and worldwide[22]; however, few of them have focused on the health burden related to short-term $PM_{2.5}$ under carbon-neutral-target scenarios. It thus remains unclear how carbon-neutral-target-related policies may impact the $PM_{2.5}$-related disease burden in China. Furthermore, due to the lack of robust exposure-response relationships,[23–31] especially regarding the association between the incidence of cardiovascular diseases and short-term $PM_{2.5}$ exposure, previous studies have mostly focused on projecting the mortality burden related to ambient $PM_{2.5}$ rather than the incidence burden. Therefore, the future trend of the incidence risk of cardiovascular diseases related to $PM_{2.5}$ is still not well understood.

Here, we take a three-step approach to project future short-term $PM_{2.5}$-associated excess incidence of cardiovascular diseases (Figure S1): first we apply a case-crossover study to estimate the exposure-response relationship between daily $PM_{2.5}$ exposure and incidence of stroke and coronary heart disease (CHD); second, we use an air quality modeling system to simulate the future daily $PM_{2.5}$ concentration from 2025 to 2060 under three emission scenarios (Table S1)—SSP245 (current-situation scenario), SSP126 (carbon-peak scenario), and SSP119 (approximate carbon-neutral scenario); and finally, we assess the short-term $PM_{2.5}$-associated excess incidence of CHD and stroke for the baseline period of 2015–2020 and future periods and explore the contribution of driving factors. We find that, compared to the 2015–2020 baseline, annual average $PM_{2.5}$ concentrations nationwide in 2060 under SSP119 (approximately carbon-neutral scenario) are projected to decrease by 81.07%. The short-term $PM_{2.5}$-related excess incidence of the two diseases will be reduced to 3,352 (95% confidence interval [CI]: 939, 5,738), far less than 34,485 (95% CI: 9,662, 59,022) under SSP245. Related healthcare expenditure is projected to reduce by more than 95% under SSP119. The continuous emission control under China's carbon-neutral policies could play a critical role in bringing large health benefits and saving healthcare expenditure.

## RESULTS

### Association between $PM_{2.5}$ and disease incidence

A total of 129,568 CHD and 143,552 stroke cases were included in this study (Figure S2). Overall, the average age of cases was around 70 years old, and the incidence was higher in males than in females (see Table 1). Our exposure data distribution presented heterogeneity in both air pollution and meteorological conditions (see Table S2; Figure S2).

Figure 1 indicated that the current day exposure of $PM_{2.5}$ (lag0) was associated with the highest increase in CHD and stroke incidence. With every 10 $\mu g/m^3$ rise in the lag0 day's $PM_{2.5}$, there was an associated increase of 0.83% (95% CI: 0.39%, 1.27%) in the acute incidence of CHD and a 0.73% (95% CI: 0.15%, 1.31%) increase in stroke incidence. Figure 1 presents a gradual decline in the associations from lag1 to lag 3, while the association between CHD incidence and exposure on lag3 day reached statistical significance, which suggested that the triggering effects of $PM_{2.5}$ exposure on CHD incidence may occur in the short term within 3 days.

### $PM_{2.5}$ concentration in baseline and future periods

Figure 2 showed that in the baseline years (2015–2020), the annual average $PM_{2.5}$ concentration in most areas of the country did not meet the WHO interim target-3 (IT-3) standard (15 $\mu g/m^3$). With the exception of the Pearl River Delta (PRD), the major urban agglomerations did not meet the IT-1 standard (35 $\mu g/m^3$). Under all the three SSP scenarios, the $PM_{2.5}$ concentration in China could show a downward trend in the future, and the areas with relatively high concentrations would still present in the Beijing-Tianjin-Hebei (BTH) region and parts of the northeastern regions. Under the most ideal SSP119 scenario (approximately carbon-neutral scenario), the average $PM_{2.5}$ in China in 2060 could drop by 81.07% compared to the baseline. Under this scenario, most counties in the southern regions can meet the AQG standard recommended by the WHO, and other regions would also be under better control. Under the SSP126 scenario (approximately carbon-peak scenario), the overall $PM_{2.5}$ concentration in the country could be close to but slightly higher than that under the SSP119 scenario, and the $PM_{2.5}$ concentration could decrease by 74.48%. Regarding the SSP245

**Table 1. Summary description on incidence data between 2013 and 2018**

| | CHD | Stroke |
|---|---|---|
| No. of cases | 129,568 | 143,552 |
| Age (mean, years old) | 70.9 | 71.9 |
| Sex (no. [%]) | | |
| Female | 57,393 (44.3) | 65,704 (45.8) |
| Male | 72,175 (55.7) | 77,846 (54.2) |
| Incidence rate (per 100,000 people) | 126.82 | 375.83 |

scenario, although the average $PM_{2.5}$ concentration in 2060 would also drop by 53.17% compared to the baseline, there could still a large gap when referring to the concentration levels under the SSP119 and SSP126 scenarios (see Tables S3 and S4; Figure S3).

### Short-term $PM_{2.5}$-related excess incidence of CHD and stroke

The annual mean excess incidence related to short-term $PM_{2.5}$ exposure was 144,672 (95% CI: 40,532, 247,607) during the baseline of 2015–2020, including 104,603 (95% CI: 21,642, 186,554) stroke and 40,068 (95% CI: 18,890, 61,052) CHD. With the dual effects of future changes in the $PM_{2.5}$ concentration and population in China, the excess risk of CHD and stroke associated with short-term $PM_{2.5}$ exposure would gradually decrease over time (Table S5). Taking the SSP2-S2 population scenario as an example, the total excess incidence of the two diseases in 2060 would be reduced to 3,352 (95% CI: 939, 5,738) and 7,188 (95% CI: 2,013, 12,301) under the SSP119 and SSP126 scenarios, respectively, far less than 34,485 (95% CI: 9,662, 59,022) under the SSP245 scenario (see Figures 3 and 4). The corresponding additional economic burdens for CHD and stroke would be reduced to 13,548.8 thousand RMB yuan (95% CI: 6,394.8, 20,659) and 73,204.8 thousand RMB yuan (95% CI: 15,130.2, 130,554.6) in SSP119, respectively, which indicated a 95.03%–97.67% decrease compared to the baseline economic burden (Table S6). The same trend was observed among five different regions. Especially for the PRD region, the total excess incidence was projected to be close to zero around the year 2045 under SSP119 (Figure S4). Figure 5 shows the spatial distribution of short-term $PM_{2.5}$-related excess incidence, which indicates that the BTH region, the most densely populated and heavily polluted region, could experience a dramatic reduction of its future excess incidence under the SSP119 and SSP126 scenarios.

### Contributors to the short-term $PM_{2.5}$-related excess incidence

Emissions and population were the two major contributors to the excess incidence, and our study found that emissions were the dominant factor. Figure 6 showed that in 2030, under all scenarios across each region, population could lead to the excess incidence increasing slightly, but the overall incidence burden could remain on a downward trend due to the large decline in $PM_{2.5}$ concentrations. By 2060, population would still increase the excess incidence but only in the PRD and the Chengdu-Chongqing (CY) regions, while population changes in the BTH, the Yangtze River Delta (YRD), and the Fenwei Plain (FW) regions would cause a small decrease. Among them, the contributions from population changes in BTH and YRD were expected to be slightly lower than those of the other regions.

## DISCUSSION

There is a need to establish a baseline relationship between cardiovascular disease and short-term exposure to $PM_{2.5}$ and to project potential changes under future warming scenarios.

We found significant associations between short-term exposure to $PM_{2.5}$ and the incidence risk of CHD and stroke, which is consistent with previous studies on the Chinese population. A 184-city time-series study reported estimated increases in the hospitalization risk associated with daily $PM_{2.5}$ exposure of 0.31% and 0.29% for CHD and ischemic stroke, respectively[32]; the results of a 172-city study based on a basic medical insurance dataset reported that the risk of hospitalization for ischemic stroke increased by 0.34% (95% CI: 0.20%, 0.48%).[33] Our estimates for the associated incidence risk of CHD and stroke were 0.83% and 0.73%, respectively, which are both higher than the results of previous national multicenter studies. Compared to the results of a meta-analysis, the estimation of the stroke incidence risk in this

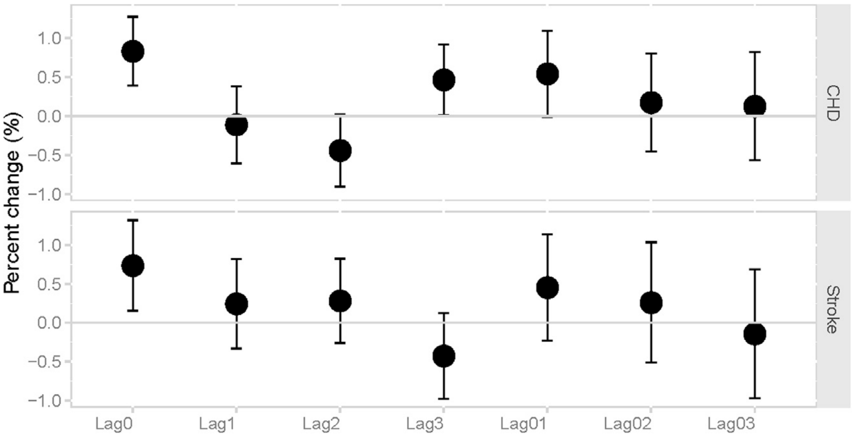


**Figure 1. Percent change in incidence risk associated with each 10 μg/m$^3$ increase of $PM_{2.5}$ on lag0**
Error bars indicate the 95% confidence intervals for the estimated percent change.

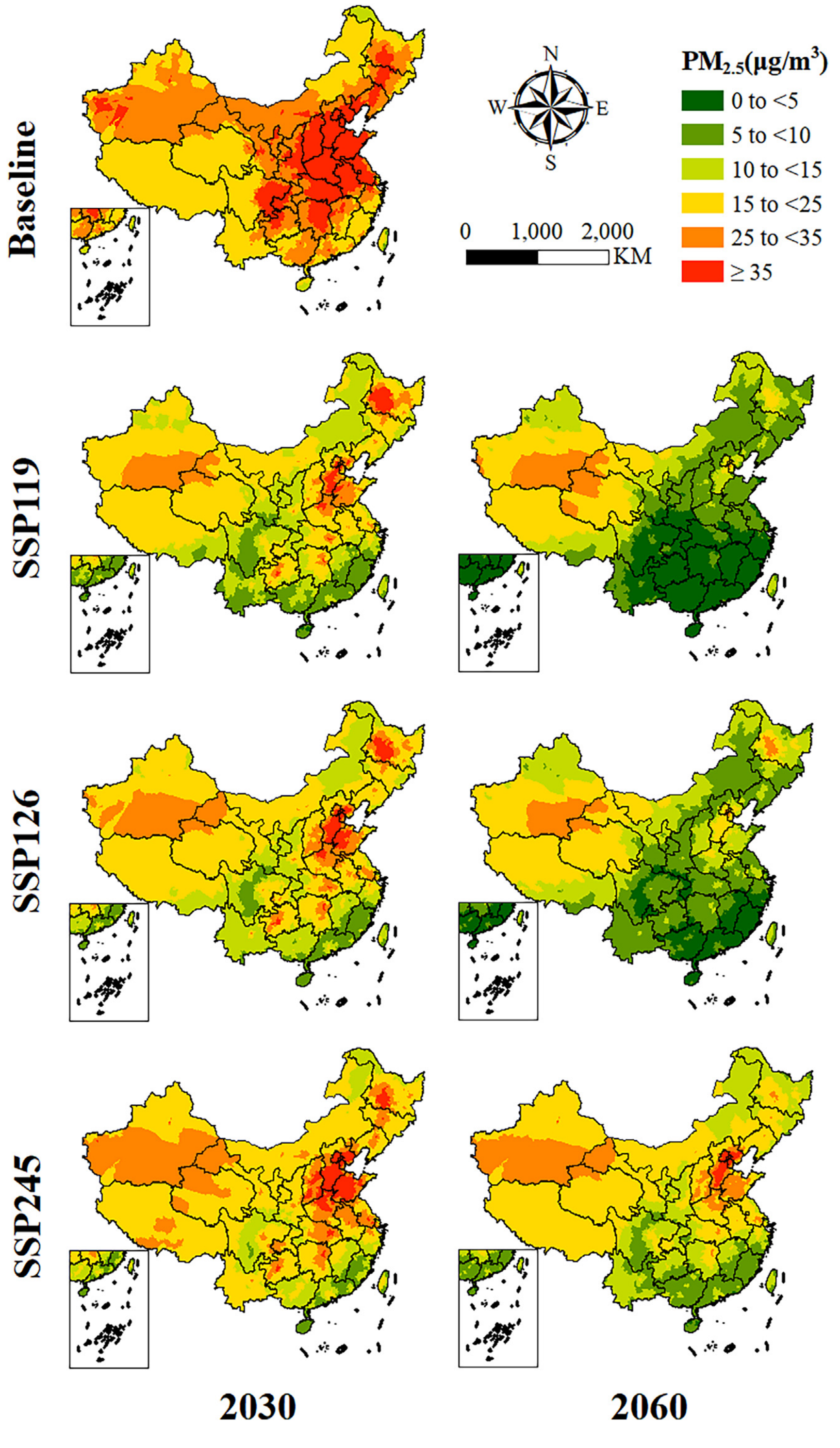


**Figure 2. Spatial distribution of annual average $PM_{2.5}$ concentration at baseline and in 2030 and 2060 under different SSP scenarios**

study is close to the pooled result of 1.1% (95% CI: 1.0%, 1.2%),[34] reflecting the consistency with the results of international studies. The application of population-representative incidence data is critical for increasing the accuracy and power of the statistical results of this study.

The short-term $PM_{2.5}$-associated excess incidence was projected to decrease in the future, especially under the approximately carbon-neutral scenario. There could be a maximum decline in the total incidence of cardiovascular diseases and related healthcare expenditures under SSP119 and a moderate decline under approximately carbon-peak scenario SSP126 and a minimum decline under current-situation scenario SSP245, implying that China's carbon-neutral policies may gain higher health as well as economic benefits. This finding is generally consistent with previous studies despite differences in the projected risk type, scenarios, regions, exposure data used, and population.[16,19,35,36] As reported, there were 47,000 avoided excess deaths in 74 Chinese cities in 2017 compared to 2013,[35] while the attributable chronic mortality could be reduced by 370,000.[36] The mitigation of greenhouse gases and pollution emission sources plays critical roles in bringing the benefits of eliminating climate change and air pollution, as well as subsequent health risk avoidance. Therefore, this study provided health-benefit-oriented policy support for China's two-carbon target. In addition, we found that stoke was projected to remain the major cardiovascular disease with a higher excess incidence than CHD, which was consistent with previous evidence and thereby revealed a necessity to pay more attention to cardiovascular diseases sensitive to $PM_{2.5}$ pollution.[37]

While the majority of regions in China are projected to experience a significant reduction in $PM_{2.5}$ concentration from 2030 to 2060 under the approximately carbon-neutral scenario, the regions most affected by $PM_{2.5}$ will persistently include the BTH, the Northeast Plain, and areas around Xinjiang Province in western China. The spatial pattern of excess incidence burden was close to the distribution of $PM_{2.5}$ concentration under the same scenarios (see Figure 2) but with a larger extent due to the expansion of future populations (refer to Figure 5). On one hand, $PM_{2.5}$ pollution in these regions could still exceed the WHO AQG levels under SSP119. According to the previous study, although there is a predicted reduction in $PM_{2.5}$ concentration ranging from 6.3–11.1 $\mu g/m^3$ compared to the baseline in a low-emission scenario (SSP126), the $PM_{2.5}$ concentration may persist above the WHO limits of 25 $\mu g/m^3$ until the

CellPress
OPEN ACCESS

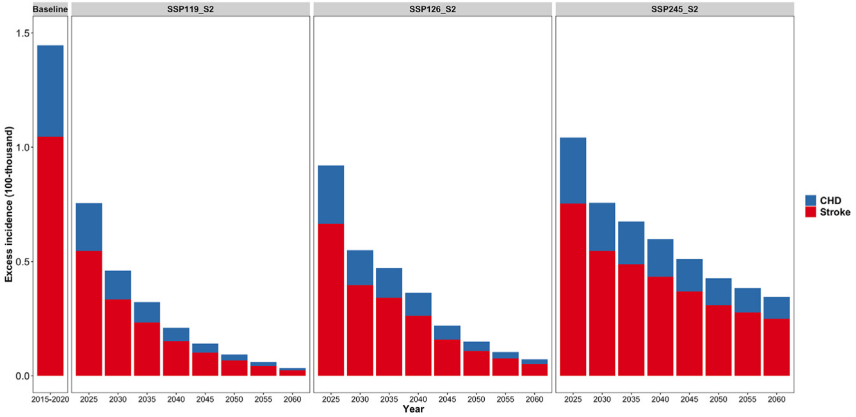


**Figure 3. Annual average excess incidence related to short-term $PM_{2.5}$ exposure at baseline and in future years under the combined scenarios of SSP2-S2 population and three SSPs**

year of 2050.[37] On the other hand, the rapid growth of the regional population under $PM_{2.5}$ exposure may offset the health benefits derived from $PM_{2.5}$ emission reduction.[38] Therefore, this finding reveals the necessity for continued and targeted emission control measures in these regions.[30,39]

Future excess incidence of cardiovascular diseases related to $PM_{2.5}$ could be driven by the dual impacts from changes in emissions and population. According to the results of contribution to the change of future excess incidence nationwide and in five regions, we observed that emission reduction could play a critical role in health burden reduction, which was consistent with previous studies.[35,37] For the entire country, the impact from population growth may contribute to increase the short-term $PM_{2.5}$-associated excess incidence in 2030 but drive the reduction of the excess incidence in 2060. However, population growth could persistently contribute to increase excess incidence over time in the PRD and CY regions. Our findings suggested that emission reduction could be the most effective way to reduce the short-term $PM_{2.5}$-associated disease burden,[1,2,40] while regional differences due to population growth may require region-specific policy implementation.

This study performed a comprehensive projection of short-term $PM_{2.5}$-associated excess incidence of cardiovascular diseases by using future scenarios aligned with carbon-neutral policies. Our results contribute to a better understanding of the health benefits associated with China's carbon-neutral target. Meanwhile, this study also overcame the lack of exposure-response relationship between short-term exposure to $PM_{2.5}$ and incidence of cardiovascular diseases, which provided

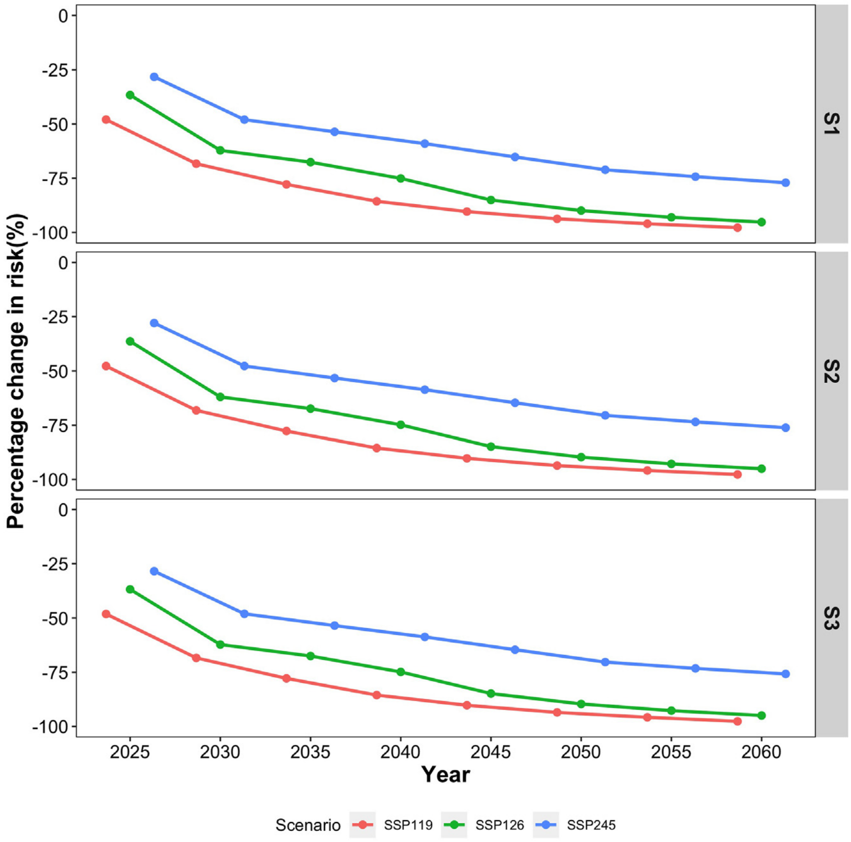


**Figure 4. Changes in the excess incidence related to short-term $PM_{2.5}$ exposure in future years under the combined scenarios of SSP2 (S1, S2, S3) population and three SSPs, as compared to the baseline**

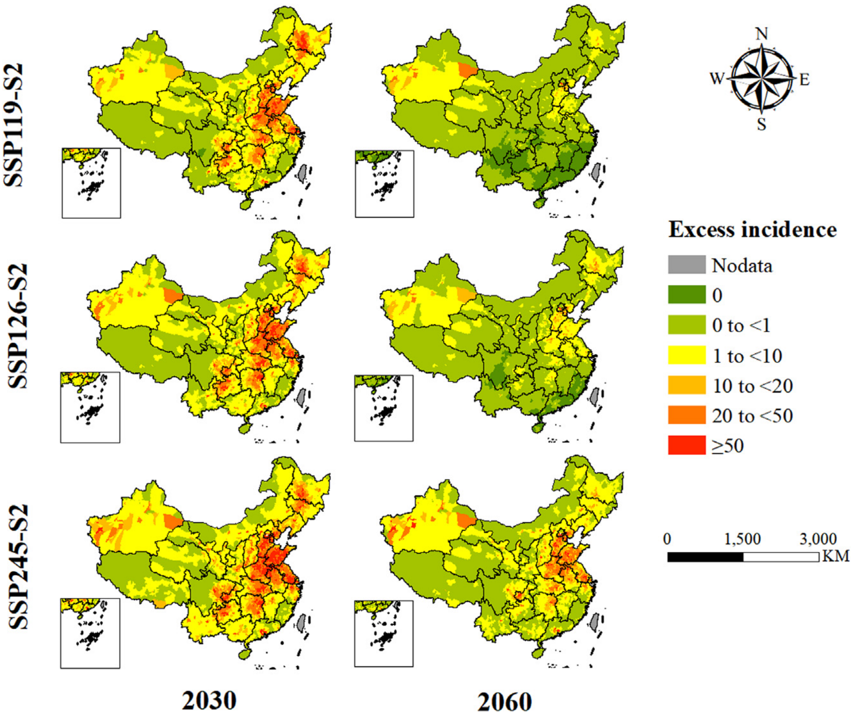


**Figure 5. Spatial distribution of the excess incidence related to short-term $PM_{2.5}$ exposure at baseline and in 2030 and 2060 under the combined scenarios of SSP2-S2 population and three SSPs**

another important term to evaluate $PM_{2.5}$-associated health burdens other than the mortality. The results of this study should be considered in light of the following limitations and uncertainties. First, the health data on cardiovascular diseases used in this study do not cover the whole country due to data limitations. This may introduce uncertainty when estimating the excess incidence.[41] Second, possible changes in the age structure are not considered, which may underestimate the excess incidence related to $PM_{2.5}$. Third, incidence rates of CHD and stroke remained unchanged in the future projection due to data unavailability, which could bring uncertainties in the estimation of excess incidence.

In summary, China's carbon-neutral policies may gain dramatic health benefits of a decreased burden of short-term $PM_{2.5}$-associated cardiovascular diseases. The implementation of the carbon-neutral target could achieve air pollution control and health benefits. Since the regions that have been densely polluted in the past will still face a higher short-term $PM_{2.5}$-associated cardiovascular disease burden in the future, targeted policy implementation should adapt to local conditions.

## EXPERIMENTAL PROCEDURES

### Resource availability

#### *Lead contact*

Further information and requests for resources should be directed to and will be fulfilled by the lead contact, Dr. Tiantian Li (litiantian@nieh.chinacdc.cn).

#### *Materials availability*

This study did not generate new unique materials.

#### *Data and code availability*

Daily $PM_{2.5}$ concentration simulation database: https://github.com/KailaiLu/PM2.5_incidence_NC. Population database: https://springernature.figshare.com/collections/Provincial_and_gridded_population_projection_for_China_under_shared_socioeconomic_pathways_from_2010_to_2100/4605713. All analyses reported in this study used the statistical software R (v.4.2.2). The code used for the main analysis is available at https://github.com/KailaiLu/PM2.5_incidence_NC. Any additional information required for reanalyzing the data reported in this paper is available from the lead contact upon reasonable request.

### Association between short-term $PM_{2.5}$ and incidence

We collected county-level individual incidence cases from 32 Chinese counties in 14 provinces between 2013 and 2018 from the Chinese Environmental Public Health Tracking system.[42] The International Classification of Diseases, Tenth Revision (ICD-10), was adopted to identify two causes: CHD (I20-I25) and stroke (I60-I64).

We conducted a time-stratified case-crossover study to obtain the associations between CHD or stroke incidence and short-term exposure to $PM_{2.5}$. In this study, the case days were the dates of disease incidence; the control days were restricted to days falling on the same day of the week, month, and year as the case days to control for time-varying and time-invariant individual confounders. Using this time-stratified approach, each case day could be matched with 3–4 control days. We then fitted a conditional logistic regression model to obtain the exposure-response relationship between $PM_{2.5}$ and incidence of CHD or stroke.[43] The regression model incorporated county-level daily average $PM_{2.5}$ as a main effect and controlled several covariates including (1) a smoothing function using natural splines (ns) with three degrees of freedom (df) for both the daily temperature and the relative humidity in the model and (2) a dichotomous variable for adjusting the holiday effects (1 = holiday; 0 = non-holiday). To investigate the delayed effect of $PM_{2.5}$, we separately fitted the models considering different lags up to 3 days prior to the date of incidence, including single-day lags (lag1, lag2, lag3) and cumulative-day lags (lag0-1, lag0-2, lag0-3). We reported the odds ratio (OR) and 95% CI to indicate the association between daily $PM_{2.5}$ and the incidence of CHD and stroke.

### Predicting future $PM_{2.5}$ concentrations

We applied China's future anthropogenic emission datasets from the dynamic projection model for emissions in China (DPEC)[7] to simulate future $PM_{2.5}$ concentrations in this work. The DPEC model contains a series of Chinese emission pathways from 2015 to 2060 under a range of socioeconomic, climate, and pollution control policies,[7] and three scenarios, namely SSP119, SSP126, and SSP245, were selected to represent the impacts of China's carbon-neutrality policy, carbon-peak policy, and current situation, respectively.

We then utilized the offline coupled Weather Research and Forecasting (WRF; v.3.9.1)-Community Multiscale Air Quality (CMAQ; v.5.2) air quality modeling system to obtain the variations in future $PM_{2.5}$ concentrations by 2060 under the three scenarios above. The air quality modeling covered mainland China with a spatial resolution of 36 km and a vertical resolution of 14 layers. Each scenario was simulated for the whole year with a 1-month spin up. Meteorological inputs were provided by WRF simulations and were fixed to the base-year (i.e., 2015) level. For emission inputs, China's historical and future anthropogenic emissions were derived from the MEIC[44] and DPEC[45] models, respectively; data for neighboring countries were obtained from the MIX[46] and CMIP6[47] databases, respectively. Natural source emissions, including biogenic (from MEGANv.2.1[48]), open biomass burning (from the

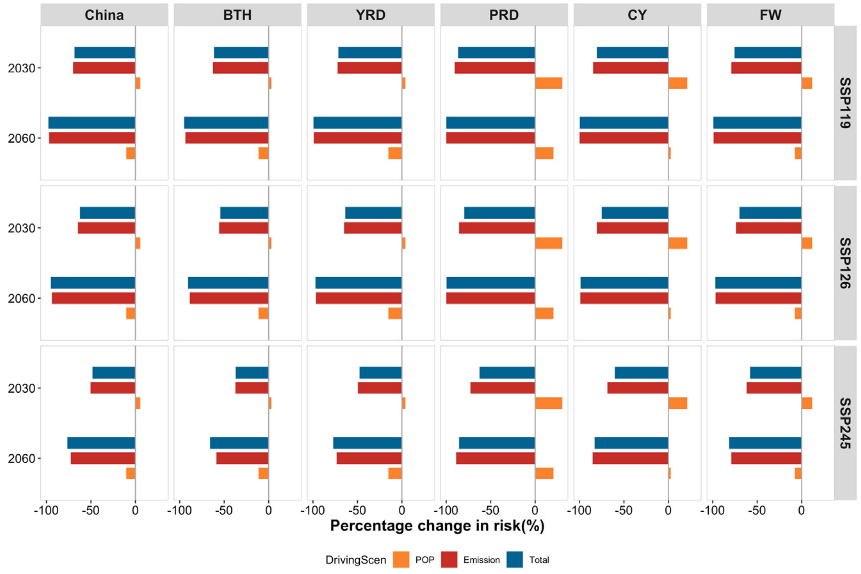


**Figure 6. Contribution rate of emission and population in changes of excess incidence using medium population under SSP scenarios**

GFED4 database[49]), and dust emissions (online simulation) were all included. The initial and boundary chemical conditions were dynamically provided by GEOS-Chem simulations. Detailed above-mentioned configurations, as well as meteorology parameterizations and chemical mechanism, are documented in Table S1.

Furthermore, to reduce the uncertainty introduced by the chemical transport model, we used observation-based $PM_{2.5}$ hindcast datasets to calibrate the daily WRF-CAMQ simulation by grid as follows:

$$C_{i,j} = C_{TAP2015} \times \frac{C_{SIM\,i,j}}{C_{SIM\,base,2015}} \quad \text{(Equation 1)}$$

where $C_{i,j}$ represents the predicted $PM_{2.5}$ concentration of scenario $i$ in year $j$. $C_{TAP2015}$ is the base-year (i.e., 2015) $PM_{2.5}$ hindcast concentration. $C_{SIM\,i,j}$ and $C_{SIM\,base,2015}$ represent the WRF-CMAQ-simulated $PM_{2.5}$ concentrations of scenario $i$ in year $j$ and of the base year, respectively.

## Incidence rate and population projection

The nation-level baseline incidence rates of CHD and stroke were calculated by dividing the sum of the annual average cases by the total census population of the 32 counties from 2013 to 2018. We assumed that the baseline rate would remain stable in the future. We matched the population data for the year corresponding to the $PM_{2.5}$ exposure data. We applied three scenarios under the SSP2 population scenario, including S1 (low fertility rate), S2 (medium fertility rate), and S3 (high fertility rate). The gridded SSP2 population at a 1 × 1 km resolution was from previous work[50] in which the future yearly Chinese population was extracted and reanalyzed based on a global population projection by considering recent fertility-promoting policies implemented in China. The gridded population was calculated at the county level by summing the population in each grid within the county.

## Projection of short-term $PM_{2.5}$-associated excess incidence

Based on the data presented above, we computed the daily excess incidence related to short-term $PM_{2.5}$ exposure for each county as follows:

$$\Delta Incidence_i = \sum_{j=1,\ldots,n} POP_i \times Y \times AF \times (C_{ij} - C_0) \quad \text{(Equation 2)}$$

where $\Delta Incidence_i$ represents the daily excess incidence associated to short-term $PM_{2.5}$ exposure for county $i$ on day $j$ during each year; $POP_i$ is the total population of county $i$ in the corresponding year; $Y$ is the incidence rate of each disease; $AF$ is the attributable fraction of stroke or CHD incidence associated with short-term $PM_{2.5}$ exposure, which was calculated using (OR-1)/OR derived from the results of the exposure-response relationship analysis (we assumed that the $AF$ was nationally consistent and stable in the future); $C_{ij}$ is the county-level daily average $PM_{2.5}$ concentration in county $i$ on day $j$; and $C_0$ is the AQG level of daily average $PM_{2.5}$ concentration set at 15 $\mu g/m^3$, which is an evidence-informed derivation of the AQG level recommended by the WHO in 2021. Therefore, the associated excess incidence can only be calculated when $C_{ij}$ exceeds $C_0$. We first calculated the daily level, county-specific $PM_{2.5}$-associated excess incidence of stroke and CHD across the country from 2015 to 2020 as the baseline, and we projected the excess incidence from 2025 up to 2060 at 5-year intervals (namely 2025, 2030, 2035, 2040, 2045, 2050, 2055, and 2060) under the combined scenarios of three $PM_{2.5}$ emission control scenarios (SSP119, SSP126, and SSP245) and three SSP2 population scenarios (S1, S2, and S3) to predict the diversity of future changes. Following this, we computed the annual average excess incidence related to short-term $PM_{2.5}$ in each county for the baseline and for each projection year. Subsequently, we aggregated the annual excess incidence number in each county according to different geographical extents including the entire country and five city agglomerations including the BTH, the YRD, the PRD, the FW, and the CY regions.

Moreover, to evaluate the potential economic burden related to the excess incidence of CHD and stroke, we computed the annual mean additional expenditure for both baseline and future years across various scenarios. We utilized the average per-hospitalization costs for CHD (14,600 RMB yuan) and stroke (30,200 RMB yuan), published in the Report on Cardiovascular Health and Diseases in China, serving as a proxy for the overall expenditure of disease treating.[51] Then, we calculated the additional expenditure by combining the excess incidence numbers with the individual costs for CHD and stroke, respectively. Following that, we compared the annual mean additional expenditure in future years across various scenarios to that of baseline to quantify the economic benefits.

To explore the contribution to future incidence changes from the impact of population and emission control, we set up three virtual scenarios. During this phase, we set a new baseline, which engaged population in 2020 and daily average $PM_{2.5}$ concentration from 2015 to 2020. The results of this new baseline may be very close to the previous baseline, but to calculate the impact of the two factors more accurately, we use this baseline to exclude errors caused by different calculation methods, and the new baseline was used only for calculating the contribution of each driving factor. Then, we set up the population scenario (POP) and the emission scenario (emission). The POP scenario used the same $PM_{2.5}$ concentration data as the new baseline scenario and the SSP2-S2 future population, while the emission scenario used the 2020 population, consistent with the new baseline scenario with future $PM_{2.5}$ concentrations under SSP119, SSP126, and SSP245. The results could represent the respective contributions from the changes in $PM_{2.5}$ exposure and population. Using the same method, we also conducted regional analysis on the five key regions (BTH, YRD, PRD, CY, and FW) in China.

All analyses were conducted in R 4.0.3 (R Foundation for Statistical Computing, Vienna, Austria) and ArcGIS 10.2 (ESRI, Redlands, CA, USA).

## SUPPLEMENTAL INFORMATION

Supplemental information can be found online at https://doi.org/10.1016/j.oneear.2024.01.006.

## ACKNOWLEDGMENTS

This work was supported by the National Natural Science Foundation of China (82241051, 82204001, and 92143202), the Open Fund by the Jiangsu Key Laboratory of Atmospheric Environment Monitoring and Pollution Control (KHK2108), the National Key Research and Development Program of China (2020YFA0607804), and the Wellcome Trust (209734/Z/17/Z). The funders of the study were not involved in study design, data collection, data analysis, data interpretation, or writing of the report.

## AUTHOR CONTRIBUTIONS

T.L., J.B., and J. Hao contributed to the study design and coordination. T.L., J. Hao, J. Hu, W.C., Z.L., P.G., Y.L., and X.G. contributed to study supervision. J.C., D.T., J.B., K.L., Y.L., Y.C., C.W., and Z.Z. contributed to data processing. J.B., J.C., C.Z., and K.L. performed the analysis. J.B., J.C., and C.Z. drafted the manuscript. J.B., J.C., D.T., and T.L. contributed to manuscript review and revision. All authors were responsible for the decision to submit the manuscript.

## DECLARATION OF INTERESTS

The authors declare no competing interests.

Received: July 10, 2023
Revised: October 31, 2023
Accepted: January 8, 2024
Published: January 30, 2024

## REFERENCES

1. Cohen, A.J., Brauer, M., Burnett, R., Anderson, H.R., Frostad, J., Estep, K., Balakrishnan, K., Brunekreef, B., Dandona, L., Dandona, R., et al. (2017). Estimates and 25-year trends of the global burden of disease attributable to ambient air pollution: an analysis of data from the Global Burden of Diseases Study 2015. Lancet *389*, 1907–1918.
2. Organization, W.H. (2016). Ambient Air Pollution: A Global Assessment of Exposure and Burden of Disease.
3. Health Effects Institute (2020). State of Global Air 2020 (Health Effects Institute).
4. Scovronick, N., Budolfson, M., Dennig, F., Errickson, F., Fleurbaey, M., Peng, W., Socolow, R.H., Spears, D., and Wagner, F. (2019). The impact of human health co-benefits on evaluations of global climate policy. Nat. Commun. *10*, 2095.
5. Xie, Y., Dai, H., Xu, X., Fujimori, S., Hasegawa, T., Yi, K., Masui, T., and Kurata, G. (2018). Co-benefits of climate mitigation on air quality and human health in Asian countries. Environ. Int. *119*, 309–318.
6. Tong, D., Geng, G., Jiang, K., Cheng, J., Zheng, Y., Hong, C., Yan, L., Zhang, Y., Chen, X., Bo, Y., et al. (2019). Energy and emission pathways towards PM2.5 air quality attainment in the Beijing-Tianjin-Hebei region by 2030. Sci. Total Environ. *692*, 361–370.
7. Cheng, J., Tong, D., Zhang, Q., Liu, Y., Lei, Y., Yan, G., Yan, L., Yu, S., Cui, R.Y., Clarke, L., et al. (2021). Pathways of China's PM2.5 air quality 2015-2060 in the context of carbon neutrality. Natl. Sci. Rev. *8*, nwab078.
8. Feng, Y., Ning, M., Lei, Y., Sun, Y., Liu, W., and Wang, J. (2019). Defending blue sky in China: Effectiveness of the "Air Pollution Prevention and Control Action Plan" on air quality improvements from 2013 to 2017. J. Environ. Manag. *252*, 109603.
9. Xue, T., Liu, J., Zhang, Q., Geng, G., Zheng, Y., Tong, D., Liu, Z., Guan, D., Bo, Y., Zhu, T., et al. (2019). Rapid improvement of PM2. 5 pollution and associated health benefits in China during 2013–2017. Sci. China Earth Sci. *62*, 1847–1856.
10. Yue, H., He, C., Huang, Q., Yin, D., and Bryan, B.A. (2020). Stronger policy required to substantially reduce deaths from PM2.5 pollution in China. Nat. Commun. *11*, 1462.
11. Kinney, P.L. (2018). Interactions of Climate Change, Air Pollution, and Human Health. Curr. Environ. Health Rep. *5*, 179–186.
12. Shindell, D., and Smith, C.J. (2019). Climate and air-quality benefits of a realistic phase-out of fossil fuels. Nature *573*, 408–411.
13. Roth, G.A., Mensah, G.A., Johnson, C.O., Addolorato, G., Ammirati, E., Baddour, L.M., Barengo, N.C., Beaton, A.Z., Benjamin, E.J., Benziger, C.P., et al. (2020). Global Burden of Cardiovascular Diseases and Risk Factors, 1990-2019: Update From the GBD 2019 Study. J. Am. Coll. Cardiol. *76*, 2982–3021.
14. Chen, W.W., Gao, R.L., Liu, L.S., Zhu, M.L., Wang, W., Wang, Y.J., Wu, Z.S., Li, H.J., Gu, D.F., Yang, Y.J., et al. (2017). China cardiovascular diseases report 2015: a summary. J. Geriatr. Cardiol. *14*, 1–10.
15. Zhao, D., Liu, J., Wang, M., Zhang, X., and Zhou, M. (2019). Epidemiology of cardiovascular disease in China: current features and implications. Nat. Rev. Cardiol. *16*, 203–212.
16. Liu, H., Liu, J., Li, M., Gou, P., and Cheng, Y. (2022). Assessing the evolution of PM2.5 and related health impacts resulting from air quality policies in China. Environ. Impact Assess. Rev. *93*, 106727.
17. Li, N., Chen, W., Rafaj, P., Kiesewetter, G., Schöpp, W., Wang, H., Zhang, H., Krey, V., and Riahi, K. (2019). Air Quality Improvement Co-benefits of Low-Carbon Pathways toward Well Below the 2 degrees C Climate Target in China. Environ. Sci. Technol. *53*, 5576–5584.
18. Cai, S., Ma, Q., Wang, S., Zhao, B., Brauer, M., Cohen, A., Martin, R.V., Zhang, Q., Li, Q., Wang, Y., et al. (2018). Impact of air pollution control policies on future PM2.5 concentrations and their source contributions in China. J. Environ. Manag. *227*, 124–133.
19. Wang, Y., Hu, j., Zhu, J., Li, J., Qin, M., Liao, H., Chen, K., and Wang, M. (2021). Health Burden and economic impacts attributed to PM2.5 and O3 in china from 2010 to 2050 under different representative concentration pathway scenarios. Resour. Conserv. Recycl. *173*, 105731.
20. Xu, J., Yao, M., Wu, W., Qiao, X., Zhang, H., Wang, P., Yang, X., Zhao, X., and Zhang, J. (2021). Estimation of ambient PM(2.5)-related mortality burden in China by 2030 under climate and population change scenarios: A modeling study. Environ. Int. *156*, 106733.
21. Wang, Y., Hu, J., Huang, L., Li, T., Yue, X., Xie, X., Liao, H., Chen, K., and Wang, M. (2022). Projecting future health burden associated with exposure to ambient PM(2.5) and ozone in China under different climate scenarios. Environ. Int. *169*, 107542.
22. Im, U., Bauer, S.E., Frohn, L.M., Geels, C., Tsigaridis, K., and Brandt, J. (2023). Present-day and future PM2. 5 and O3-related global and regional premature mortality in the EVAv6. 0 health impact assessment model. Environ. Res. *216*, 114702.
23. Tagaris, E., Liao, K.J., Delucia, A.J., Deck, L., Amar, P., and Russell, A.G. (2009). Potential impact of climate change on air pollution-related human health effects. Environ. Sci. Technol. *43*, 4979–4988.
24. Tainio, M., Juda-Rezler, K., Reizer, M., Warchałowski, A., Trapp, W., and Skotak, K. (2012). Future climate and adverse health effects caused by fine particulate matter air pollution: case study for Poland. Reg. Environ. Change *13*, 705–715.
25. Fang, Y., Mauzerall, D.L., Liu, J., Fiore, A.M., and Horowitz, L.W. (2013). Impacts of 21st century climate change on global air pollution-related premature mortality. Climatic Change *121*, 239–253.
26. Madaniyazi, L., Nagashima, T., Guo, Y., Yu, W., and Tong, S. (2015). Projecting Fine Particulate Matter-Related Mortality in East China. Environ. Sci. Technol. *49*, 11141–11150.
27. Sun, J., Fu, J.S., Huang, K., and Gao, Y. (2015). Estimation of future PM2.5- and ozone-related mortality over the continental United States in a changing climate: An application of high-resolution dynamical downscaling technique. J. Air Waste Manag. Assoc. *65*, 611–623.
28. Silva, R.A., West, J.J., Lamarque, J.F., Shindell, D.T., Collins, W.J., Dalsoren, S., Faluvegi, G., Folberth, G., Horowitz, L.W., Nagashima, T., et al. (2016). The effect of future ambient air pollution on human premature mortality to 2100 using output from the ACCMIP model ensemble. Atmos. Chem. Phys. *16*, 9847–9862.

CellPress
OPEN ACCESS

29. Silva, R.A., West, J.J., Lamarque, J.F., Shindell, D.T., Collins, W.J., Faluvegi, G., Folberth, G.A., Horowitz, L.W., Nagashima, T., Naik, V., et al. (2017). Future Global Mortality from Changes in Air Pollution Attributable to Climate Change. Nat. Clim. Change *7*, 647–651.
30. Park, S., Allen, R.J., and Lim, C.H. (2020). A likely increase in fine particulate matter and premature mortality under future climate change. Air Qual. Atmos. Health *13*, 143–151.
31. Fann, N.L., Nolte, C.G., Sarofim, M.C., Martinich, J., and Nassikas, N.J. (2021). Associations Between Simulated Future Changes in Climate, Air Quality, and Human Health. JAMA Netw. Open *4*, e2032064.
32. Tian, Y., Liu, H., Wu, Y., Si, Y., Song, J., Cao, Y., Li, M., Wu, Y., Wang, X., and Chen, L. (2019). Association between ambient fine particulate pollution and hospital admissions for cause specific cardiovascular disease: time series study in 184 major Chinese cities. BMJ *367*.
33. Tian, Y., Liu, H., Zhao, Z., Xiang, X., Li, M., Juan, J., Song, J., Cao, Y., Wang, X., Chen, L., et al. (2018). Association between ambient air pollution and daily hospital admissions for ischemic stroke: a nationwide time-series analysis. PLoS Med. *15*, e1002668.
34. Shah, A.S.V., Lee, K.K., McAllister, D.A., Hunter, A., Nair, H., Whiteley, W., Langrish, J.P., Newby, D.E., and Mills, N.L. (2015). Short term exposure to air pollution and stroke: systematic review and meta-analysis. BMJ *350*, h1295.
35. Huang, J., Pan, X., Guo, X., and Li, G. (2018). Health impact of China's Air Pollution Prevention and Control Action Plan: an analysis of national air quality monitoring and mortality data. Lancet Planet. Health *2*, e313–e323.
36. Zhang, Q., Zheng, Y., Tong, D., Shao, M., Wang, S., Zhang, Y., Xu, X., Wang, J., He, H., Liu, W., et al. (2019). Drivers of improved PM2.5 air quality in China from 2013 to 2017. Proc. Natl. Acad. Sci. USAUSA *116*, 24463–24469.
37. Wang, Y., Hu, J., Zhu, J., Li, J., Qin, M., Liao, H., Chen, K., and Wang, M. (2021). Health Burden and economic impacts attributed to PM2. 5 and O3 in China from 2010 to 2050 under different representative concentration pathway scenarios. Resour. Conserv. Recycl. *173*, 105731.
38. Lavell, A., Oppenheimer, M., Diop, C., Hess, J., Lempert, R., Li, J., and Myeong, S. (2012). Managing the risks of extreme events and disasters to advance climate change adaptation. In A Special Report of Working Groups I and II of the Intergovernmental Panel on Climate Change (IPCC), pp. 25–64.
39. Zhang, Y. (2021). All-Cause Mortality Risk and Attributable Deaths Associated with Long-Term Exposure to Ambient PM2.5 in Chinese Adults. Environ. Sci. Technol. *55*, 6116–6127.
40. GBD 2016 Risk Factors Collaborators, Afshin, A., Abajobir, A.A., Abate, K.H., Abbafati, C., Abbas, K.M., Abd-Allah, F., Abdulle, A.M., Abera, S.F., and Aboyans, V. (2017). Global, regional, and national comparative risk assessment of 84 behavioural, environmental and occupational, and metabolic risks or clusters of risks, 1990–2016: a systematic analysis for the Global Burden of Disease Study 2016. Lancet *390*, 1345–1422.
41. Zhong, M., Chen, F., and Saikawa, E. (2019). Sensitivity of projected PM2. 5-and O3-related health impacts to model inputs: A case study in mainland China. Environ. Int. *123*, 256–264.
42. Ban, J., Du, Z., Wang, Q., Ma, R., Zhou, Y., and Li, T. (2019). Environmental Health Indicators for China: Data Resources for Chinese Environmental Public Health Tracking. Environ. Health Perspect. *127*, 44501.
43. Ban, J., Ma, R., Zhang, Y., and Li, T. (2021). PM2.5-associated risk for cardiovascular hospital admission and related economic burdens in Beijing, China. Sci. Total Environ. *799*, 149445.
44. Zhang, Q., Streets, D.G., Carmichael, G.R., He, K.B., Huo, H., Kannari, A., Klimont, Z., Park, I.S., Reddy, S., Fu, J.S., et al. (2009). Asian emissions in 2006 for the NASA INTEX-B mission. Atmos. Chem. Phys. *9*, 5131–5153.
45. Tong, D., Cheng, J., Liu, Y., Yu, S., Yan, L., Hong, C., Qin, Y., Zhao, H., Zheng, Y., Geng, G., et al. (2020). Dynamic projection of anthropogenic emissions in China: methodology and 2015–2050 emission pathways under a range of socio-economic, climate policy, and pollution control scenarios. Atmos. Chem. Phys. *20*, 5729–5757.
46. Li, M., Zhang, Q., Kurokawa, J.-i., Woo, J.-H., He, K., Lu, Z., Ohara, T., Song, Y., Streets, D.G., Carmichael, G.R., et al. (2017). MIX: a mosaic Asian anthropogenic emission inventory under the international collaboration framework of the MICS-Asia and HTAP. Atmos. Chem. Phys. *17*, 935–963.
47. Feng, L., Smith, S.J., Braun, C., Crippa, M., Gidden, M.J., Hoesly, R., Klimont, Z., Van Marle, M., Van Den Berg, M., and Van Der Werf, G.R. (2020). The generation of gridded emissions data for CMIP6. Geosci. Model Dev. (GMD) *13*, 461–482.
48. Guenther, A.B., Jiang, X., Heald, C.L., Sakulyanontvittaya, T., Duhl, T., Emmons, L.K., and Wang, X. (2012). The Model of Emissions of Gases and Aerosols from Nature version 2.1 (MEGAN2. 1): an extended and updated framework for modeling biogenic emissions. Geosci. Model Dev. (GMD) *5*, 1471–1492.
49. Van Der Werf, G.R., Randerson, J.T., Giglio, L., Van Leeuwen, T.T., Chen, Y., Rogers, B.M., Mu, M., van Marle, M.J.E., Morton, D.C., Collatz, G.J., et al. (2017). Global fire emissions estimates during 1997–2016. Earth Syst. Sci. Data *9*, 697–720.
50. Chen, Y., Guo, F., Wang, J., Cai, W., Wang, C., and Wang, K. (2020). Provincial and gridded population projection for China under shared socioeconomic pathways from 2010 to 2100. Sci. Data *7*, 83.
51. MA, L., WANG, Z., FAN, J., and HU, S. (2023). Interpretation of Report on Cardiovascular Health and Diseases in China 2022. Chinese General Practice *26*, 3975.

One Earth, Volume *7*

## Supplemental information

# China's carbon-neutral policies will reduce short-term $PM_{2.5}$-associated excess incidence of cardiovascular diseases

**Jie Ban, Jing Cheng, Can Zhang, Kailai Lu, Zhen Zhou, Zhao Liu, Yidan Chen, Can Wang, Wenjia Cai, Peng Gong, Yong Luo, Dan Tong, Jianlin Hu, Xinbiao Guo, Junwei Hao, and Tiantian Li**

**Supplementary**

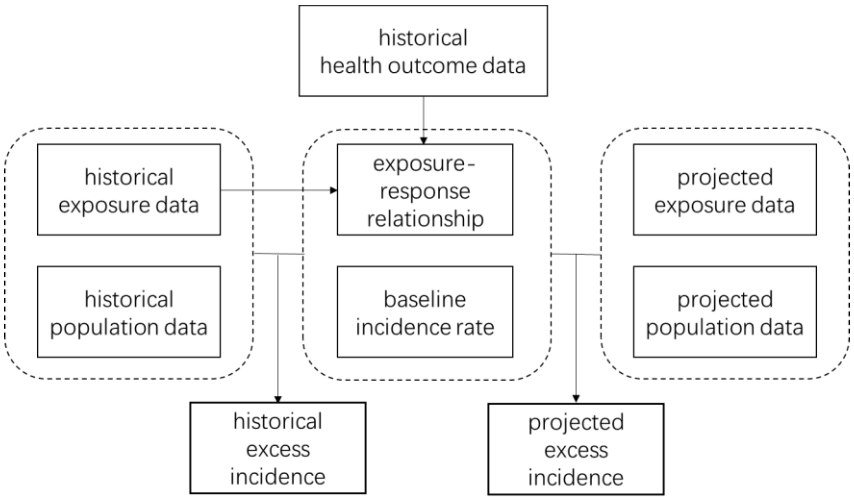


**Figure S1. Framework of integrated analysis process**

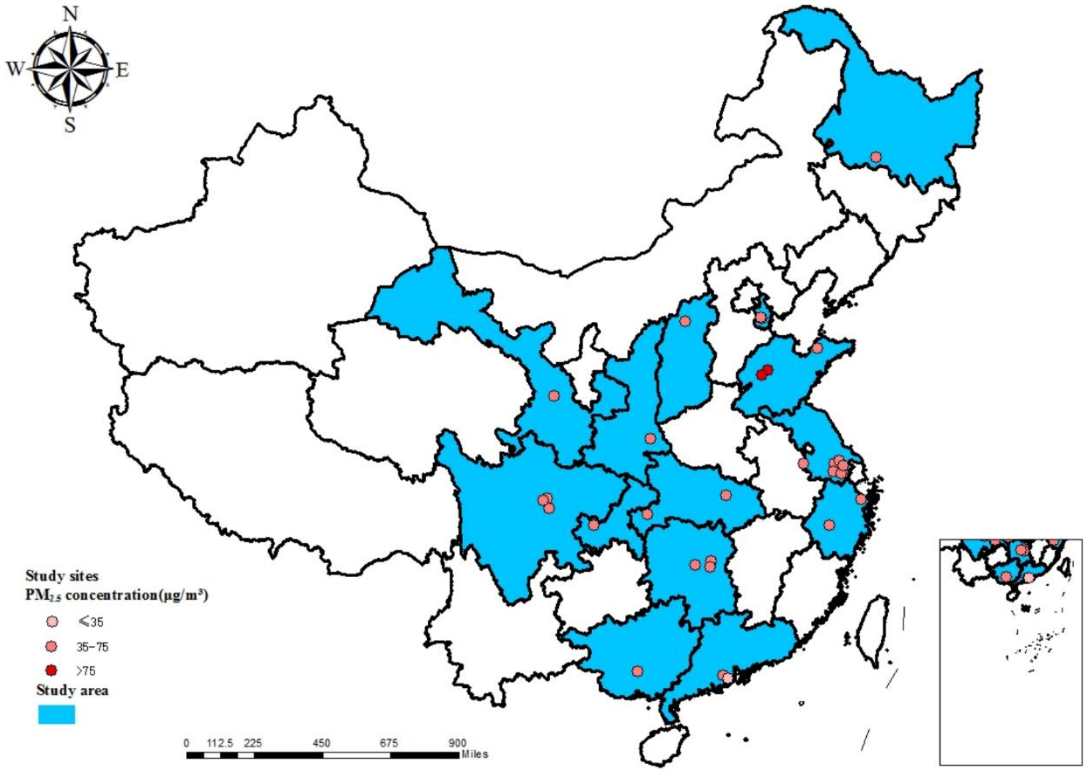


**Figure S2. Map of studied counties**

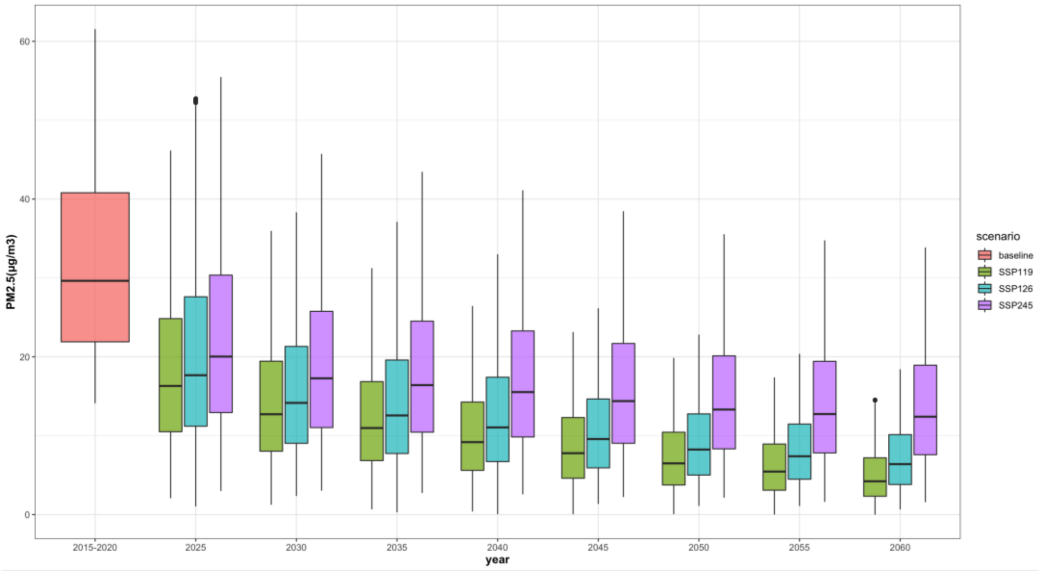


**Figure S3. Distribution of county-level annual mean $PM_{2.5}$ concentration under different emission scenarios in each year. Error bars represent the minimum and maximum concentration values.**

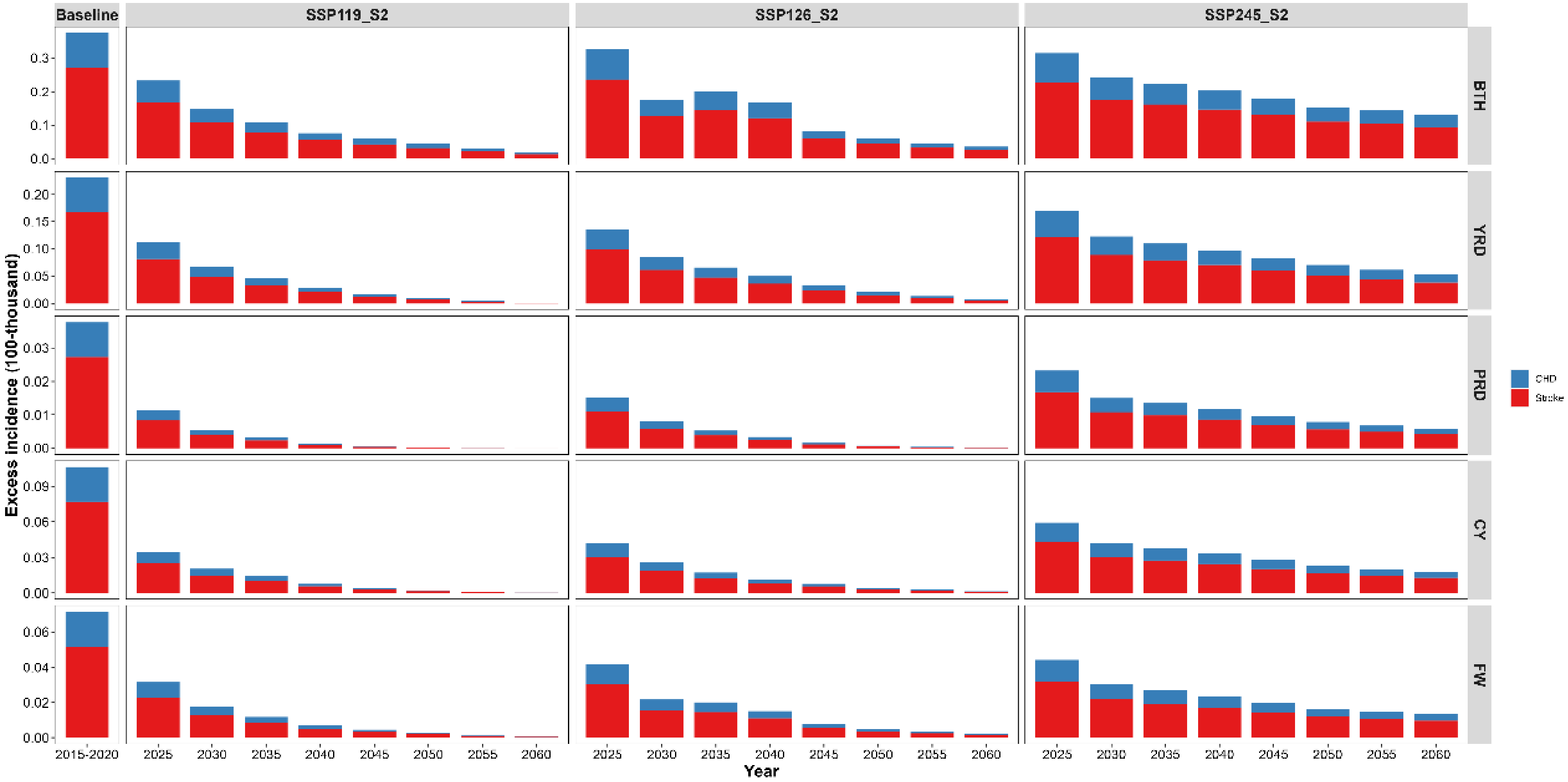


**Figure S4. Annual mean excess incidence of CHD and stroke in each region under the combined scenarios of SSP2-S2 population and three SSPs**

**Table S1. WRF-CMAQ modelling system configurations**

| Model | Parameter | Configuration schemes |
|---|---|---|
| WRF | Horizontal resolution | 36km, 181 (column) × 136 (row) |
| | Vertical resolution | 28 sigma levels from surface to tropopause |
| | Simulation period | The whole year with one-month spin-up (All CMAQ simulations applied the WRF meteorology fixed in 2015) |
| | IC and BC | Final analysis data from NCEP |
| | Shortwave radiation | New Goddard scheme |
| | Longwave radiation | RRTM scheme |
| | Surface layer | Pleim–Xiu scheme |
| | Planetary boundary layer | ACM2 scheme |
| | Cumulus | Kain–Fritsch scheme |
| | Cloud microphysics | WSM6 scheme |
| CMAQ | Horizontal resolution | 36km, 172 (column) × 127 (row) |
| | Vertical resolution | 14 sigma levels from surface to tropopause |
| | Simulation period | The whole year with one-month spin-up |
| | IC and BC | Dynamic GEOS-Chem global simulation outputs |
| | Gas-phase mechanism | CB05 |
| | Aqueous-phase mechanism | RADM |
| | Aerosol module | AERO6 |
| | Cloud module | ACM_AE6 ACM cloud processor |
| | Photolytic rate | In-line calculation |
| | Anthropogenic emissions | Historical: MEIC for China; MIX for other Asian countries<br>Future: DPEC for China; CMIP6 for other Asian countries |
| | Biogenic emissions | MEGANv2.1 |
| | Open biomass burning | GFED4 |
| | Dust | In-line calculation |
| | Lightning | Not included |

**Table S2. Summary description on environmental exposure**

| | Mean | Min | P25 | P75 | Max |
|---|---|---|---|---|---|
| $PM_{2.5}$ (μg/m$^3$) | 53.4 | 3.0 | 26.5 | 67.2 | 647.8 |
| $O_3$-8h (μg/m$^3$) | 89.5 | 1.0 | 53.6 | 117.6 | 637.0 |
| Temperature (℃) | 16.0 | -31.5 | 8.8 | 23.9 | 35.8 |
| Relative humidity (%) | 71.0 | 11.3 | 62.0 | 83.0 | 100.0 |

**Table S3. Statistics of $PM_{2.5}$ concentration**

| Scenario | Year | Mean(μg/m$^3$) | Stdev | Min | P50 | P75 | Max |
|---|---|---|---|---|---|---|---|
| Baseline | 2015-2020 | 37.91 | 16.08 | 1.01 | 34.46 | 48.41 | 85.54 |
| SSP119 | 2025 | 24.16 | 11.75 | 0.73 | 21.16 | 30.90 | 82.78 |
| | 2030 | 18.65 | 9.04 | 0.60 | 16.67 | 23.61 | 73.12 |
| | 2035 | 15.99 | 7.91 | 0.53 | 14.33 | 20.05 | 72.44 |
| | 2040 | 13.45 | 6.78 | 0.45 | 12.24 | 16.77 | 62.64 |
| | 2045 | 11.63 | 6.17 | 0.39 | 10.56 | 14.65 | 54.40 |
| | 2050 | 9.98 | 5.42 | 0.34 | 9.05 | 12.90 | 40.71 |
| | 2055 | 8.68 | 5.00 | 0.30 | 7.80 | 11.23 | 33.47 |
| | 2060 | 7.18 | 4.55 | 0.26 | 6.27 | 9.29 | 31.06 |
| SSP126 | 2025 | 26.93 | 14.19 | 0.81 | 23.53 | 33.25 | 99.37 |
| | 2030 | 20.39 | 9.35 | 0.66 | 18.33 | 25.85 | 60.93 |
| | 2035 | 18.74 | 10.07 | 0.60 | 16.57 | 22.97 | 78.26 |
| | 2040 | 16.57 | 9.05 | 0.54 | 14.78 | 20.27 | 71.27 |
| | 2045 | 13.78 | 6.39 | 0.46 | 12.67 | 17.46 | 47.69 |
| | 2050 | 11.98 | 5.62 | 0.40 | 11.10 | 15.22 | 41.24 |
| | 2055 | 10.76 | 5.02 | 0.36 | 9.97 | 13.54 | 35.92 |
| | 2060 | 9.56 | 4.82 | 0.31 | 8.79 | 12.18 | 33.50 |
| SSP245 | 2025 | 29.29 | 13.69 | 0.91 | 26.33 | 37.23 | 96.30 |
| | 2030 | 24.43 | 11.03 | 0.79 | 22.39 | 30.48 | 80.27 |
| | 2035 | 23.15 | 10.52 | 0.74 | 21.25 | 28.78 | 81.55 |
| | 2040 | 21.89 | 9.98 | 0.70 | 20.09 | 27.22 | 79.10 |
| | 2045 | 20.38 | 9.41 | 0.66 | 18.81 | 25.49 | 75.12 |
| | 2050 | 18.87 | 8.70 | 0.62 | 17.51 | 23.78 | 68.12 |
| | 2055 | 18.23 | 8.76 | 0.59 | 16.86 | 22.93 | 69.33 |
| | 2060 | 17.75 | 8.50 | 0.57 | 16.52 | 22.32 | 67.10 |

**Table S4. Change of concentration under difference scenarios**

| Year | SSP119 | SSP126 | SSP245 |
|---|---|---|---|
| 2025 | -13.74(-36.26%) | -10.98(-28.96%) | -8.61(-22.72%) |
| 2030 | -19.26(-50.81%) | -17.52(-46.22%) | -13.48(-35.56%) |
| 2035 | -21.92(-57.83%) | -19.16(-50.55%) | -14.76(-38.94%) |
| 2040 | -24.46(-64.53%) | -21.34(-56.29%) | -16.02(-42.25%) |
| 2045 | -26.28(-69.33%) | -24.13(-63.65%) | -17.52(-46.23%) |
| 2050 | -27.92(-73.66%) | -25.92(-68.39%) | -19.03(-50.21%) |
| 2055 | -29.23(-77.10%) | -27.15(-71.62%) | -19.68(-51.92%) |
| 2060 | -30.73(-81.07%) | -28.35(-74.78%) | -20.15(-53.17%) |

**Table S5. The excess incidence of stroke and CHD under difference combined scenarios of population and three SSPs**

| Emission scenarios | Year | Stroke | | | CHD | | |
|---|---|---|---|---|---|---|---|
| | | SSP2-S1 | SSP2-S2 | SSP2-S3 | SSP2-S1 | SSP2-S2 | SSP2-S3 |
| SSP119 | 2025 | 54428 (11261, 97070) | 54653 (11308, 97471) | 54242 (11222, 96738) | 20849 (9829, 31767) | 20935 (9870, 31899) | 20777 (9795, 31659) |
| | 2030 | 33148 (6858, 59119) | 33288 (6887, 59368) | 33042 (6836, 58929) | 12698 (5986, 19347) | 12751 (6011, 19429) | 12657 (5967, 19285) |
| | 2035 | 23198 (4800, 41372) | 23322 (4825, 41594) | 23179 (4796, 41339) | 8886 (4189, 13540) | 8934 (4212, 13612) | 8879 (4186, 13529) |
| | 2040 | 15041 (3112, 26825) | 15163 (3137, 27042) | 15111 (3126, 26949) | 5762 (2716, 8779) | 5808 (2738, 8850) | 5788 (2729, 8819) |
| | 2045 | 10073 (2084, 17965) | 10205 (2111, 18200) | 10214 (2113, 18216) | 3859 (1819, 5879) | 3909 (1843, 5956) | 3913 (1845, 5962) |
| | 2050 | 6588 (1363, 11749) | 6725 (1391, 11993) | 6765 (1400, 12064) | 2524 (1190, 3845) | 2576 (1214, 3925) | 2591 (1222, 3948) |
| | 2055 | 4219 (873, 7525) | 4346 (899, 7751) | 4397 (910, 7842) | 1616 (762, 2463) | 1665 (785, 2537) | 1684 (794, 2566) |
| | 2060 | 2329 (482, 4154) | 2424 (501, 4323) | 2470 (511, 4406) | 892 (421, 1359) | 928 (438, 1415) | 946 (446, 1442) |
| SSP126 | 2025 | 66266 (13710, 118182) | 66567 (13772, 118719) | 66099 (13676, 117884) | 25383 (11967, 38676) | 25499 (12021, 38852) | 25319 (11937, 38579) |
| | 2030 | 39592 (8192, 70611) | 39770 (8228, 70927) | 39482 (8169, 70413) | 15166 (7150, 23108) | 15234 (7182, 23212) | 15124 (7130, 23044) |
| | 2035 | 33901 (7014, 60460) | 34130 (7061, 60869) | 33964 (7027, 60572) | 12986 (6122, 19786) | 13074 (6163, 19920) | 13010 (6133, 19823) |
| | 2040 | 26072 (5394, 46497) | 26329 (5447, 46957) | 26277 (5437, 46865) | 9987 (4708, 15217) | 10086 (4755, 15367) | 10066 (4745, 15337) |
| | 2045 | 15645 (3237, 27902) | 15854 (3280, 28275) | 15860 (3281, 28285) | 5993 (2825, 9131) | 6073 (2863, 9253) | 6075 (2864, 9257) |
| | 2050 | 10566 (2186, 18845) | 10783 (2231, 19232) | 10832 (2241, 19318) | 4047 (1908, 6167) | 4131 (1947, 6294) | 4149 (1956, 6322) |
| | 2055 | 7324 (1515, 13062) | 7540 (1560, 13448) | 7610 (1575, 13572) | 2806 (1323, 4275) | 2888 (1362, 4401) | 2915 (1374, 4442) |
| | 2060 | 5006 (1036, 8928) | 5197 (1075, 9268) | 5277 (1092, 9411) | 1918 (904, 2922) | 1991 (938, 3033) | 2021 (953, 3080) |
| SSP245 | 2025 | 75047 (15527, 133843) | 75384 (15597, 134444) | 74851 (15486, 133494) | 28747 (13553, 43802) | 28876 (13614, 43998) | 28672 (13517, 43687) |
| | 2030 | 54402 (11256, 97023) | 54674 (11312, 97508) | 54314 (11237, 96866) | 20839 (9824, 31752) | 20943 (9873, 31911) | 20805 (9808, 31700) |
| | 2035 | 48547 (10044, 86582) | 48874 (10112, 87164) | 48630 (10061, 86729) | 18596 (8767, 28335) | 18721 (8826, 28525) | 18628 (8782, 28383) |
| | 2040 | 42852 (8866, 76425) | 43271 (8953, 77171) | 43173 (8932, 76998) | 16415 (7739, 25011) | 16575 (7814, 25255) | 16538 (7797, 25198) |
| | 2045 | 36412 (7534, 64939) | 36943 (7643, 65885) | 36998 (7655, 65984) | 13948 (6576, 21252) | 14151 (6671, 21562) | 14172 (6681, 21594) |

| | | | | | | |
|---|---|---|---|---|---|---|
| 2050 | 30229 (6254, 53912) | 30876 (6388, 55066) | 31037 (6421, 55352) | 11579 (5459, 17643) | 11827 (5576, 18021) | 11889 (5605, 18115) |
| 2055 | 26919 (5570, 48009) | 27728 (5737, 49451) | 27993 (5792, 49925) | 10312 (4861, 15712) | 10621 (5007, 16183) | 10723 (5055, 16338) |
| 2060 | 23998 (4965, 42800) | 24934 (5159, 44469) | 25317 (5238, 45152) | 9193 (4334, 14007) | 9551 (4503, 14553) | 9698 (4572, 14777) |

**Table S6. Annual mean additional hospitalization cost related to excess incidence of two diseases under the combined SSP2-S2 population and three SSPS**

| Scenario | Year | Additional hospitalization cost (thousand RMB) | |
|---|---|---|---|
| | | CHD (95%CI) | Stroke (95%CI) |
| Baseline | 2015-2020 | 584,992.8(275,794, 891,359.2) | 3,159,011(653,588.4, 5,633,931) |
| SSP119 | 2025 | 305,651 (144,102, 465,725.4) | 1,650,520.6 (341,501.6, 2,943,624.2) |
| | 2030 | 186,164.6 (87,760.6, 283,663.4) | 1,005,297.6 (207,987.4, 1,792,913.6) |
| | 2035 | 130,436.4 (61,495.2, 198,735.2) | 704,324.4 (145,715, 1,256,138.8) |
| | 2040 | 84,796.8 (39,974.8, 129,210) | 457,922.6 (94,737.4, 816,668.4) |
| | 2045 | 57,071.4 (26,907.8, 86,957.6) | 308,191 (63,752.2, 549,640) |
| | 2050 | 37,609.6 (17,724.4, 57,305) | 203,095 (42,008.2, 362,188.6) |
| | 2055 | 24,309 (11,461, 37,040.2) | 131,249.2 (27,149.8, 234,080.2) |
| | 2060 | 13,548.8 (6,394.8, 20,659) | 73,204.8 (15,130.2, 130,554.6) |
| SSP126 | 2025 | 372,285.4 (175,506.6, 567,239.2) | 2,010,323.4 (415,914.4, 3,585,313.8) |
| | 2030 | 222,416.4 (104,857.2, 338,895.2) | 1,201,054 (248,485.6, 2,141,995.4) |
| | 2035 | 190,880.4 (89,979.8, 290,832) | 1,030,726 (213,242.2, 1,838,243.8) |
| | 2040 | 147,255.6 (69,423, 224,358.2) | 795,135.8 (164,499.4, 1,418,101.4) |
| | 2045 | 88,665.8 (41,799.8, 135,093.8) | 478,790.8 (99,056, 853,905) |
| | 2050 | 60,312.6 (28,426.2, 91,892.4) | 325,646.6 (67,376.2, 580,806.4) |
| | 2055 | 42,164.8 (19,885.2, 64,254.6) | 227,708 (47,112, 406,129.6) |
| | 2060 | 29,068.6 (13,694.8, 44,281.8) | 156.949.4 (32.465, 279.893.6) |
| SSP245 | 2025 | 421,589.6 (198,764.4, 642,370.8) | 2.276.596.8 (471.029.4, 4.060.208.8) |
| | 2030 | 305,767.8 (144,145.8, 465,900.6) | 1.651.154.8 (341.622.4, 2.944.741.6) |
| | 2035 | 273,326.6 (128,859.6, 416,465) | 1.475.994.8 (305.382.4, 2632.352.8) |

| | | |
|---|---|---|
| 2040 | 241,995 (114,084.4, 368,723) | 1,306,784.2 (270,380.6, 2,330,564.2) |
| 2045 | 206,604.6 (97,396.6, 314,805.2) | 1,115,678.6 (230,818.6, 1,989,727) |
| 2050 | 172,674.2 (81,409.6, 263,106.6) | 932,455.2 (192,917.6, 1,662,993.2) |
| 2055 | 155,066.6 (73,102.2, 236,271.8) | 837,385.6 (173,257.4, 1,493,420.2) |
| 2060 | 139,444.6 (65,743.8, 212,473.8) | 753,006.8 (155,801.8, 1,342,963.8) |
